# Cell Cycle-Dependent Rho GTPase Activity Dynamically Regulates Cancer Cell Motility and Invasion *In Vivo*

**DOI:** 10.1371/journal.pone.0083629

**Published:** 2013-12-30

**Authors:** Yoshinori Kagawa, Shinji Matsumoto, Yuji Kamioka, Koshi Mimori, Yoko Naito, Taeko Ishii, Daisuke Okuzaki, Naohiro Nishida, Sakae Maeda, Atsushi Naito, Junichi Kikuta, Keizo Nishikawa, Junichi Nishimura, Naotsugu Haraguchi, Ichiro Takemasa, Tsunekazu Mizushima, Masataka Ikeda, Hirofumi Yamamoto, Mitsugu Sekimoto, Hideshi Ishii, Yuichiro Doki, Michiyuki Matsuda, Akira Kikuchi, Masaki Mori, Masaru Ishii

**Affiliations:** 1 Department of Immunology and Cell Biology, Graduate School of Medicine, Osaka University, Suita, Osaka, Japan; 2 Laboratory of Cellular Dynamics, WPI-Immunology Frontier Research Center, Osaka University, Suita, Osaka, Japan; 3 JST, CREST, Chiyoda-ku, Tokyo, Japan; 4 Department of Gastroenterological Surgery, Graduate School of Medicine, Osaka University, Suita, Osaka, Japan; 5 Department of Molecular Biology and Biochemistry, Graduate School of Medicine, Osaka University, Suita, Osaka, Japan; 6 Department of Pathology and Biology of Diseases, Graduate School of Medicine, Kyoto University, Kyoto, Japan; 7 Department of Surgery, Medical Institute of Bioregulation, Kyushu University, Beppu, Oita, Japan; 8 DNA-chip Developmental Center for Infectious Diseases, Research Institute for Microbial Diseases, Osaka University, Suita, Osaka, Japan; Seoul National University, Republic of Korea

## Abstract

The mechanism behind the spatiotemporal control of cancer cell dynamics and its possible association with cell proliferation has not been well established. By exploiting the intravital imaging technique, we found that cancer cell motility and invasive properties were closely associated with the cell cycle. *In vivo* inoculation of human colon cancer cells bearing fluorescence ubiquitination-based cell cycle indicator (Fucci) demonstrated an unexpected phenomenon: S/G2/M cells were more motile and invasive than G1 cells. Microarray analyses showed that *Arhgap11a*, an uncharacterized Rho GTPase-activating protein (RhoGAP), was expressed in a cell-cycle-dependent fashion. Expression of ARHGAP11A in cancer cells suppressed RhoA-dependent mechanisms, such as stress fiber formation and focal adhesion, which made the cells more prone to migrate. We also demonstrated that RhoA suppression by ARHGAP11A induced augmentation of relative Rac1 activity, leading to an increase in the invasive properties. RNAi-based inhibition of Arhgap11a reduced the invasion and *in vivo* expansion of cancers. Additionally, analysis of human specimens showed the significant up-regulation of *Arhgap11a* in colon cancers, which was correlated with clinical invasion status. The present study suggests that ARHGAP11A, a cell cycle-dependent RhoGAP, is a critical regulator of cancer cell mobility and is thus a promising therapeutic target in invasive cancers.

## Introduction

Unlimited expansion due to unchecked cell cycle progression and increased penetration into the normal neighboring environment is a formidable and life-threatening aspect of cancer cells. In fact, cell cycle regulation has been a major research topic in the field of cancer cell biology.

In addition, cancer has highly dynamic properties, including invasion of surrounding tissues, infiltration of the systemic circulation, and pioneering of a new ‘niche’ for colonization far from its origin [1], [2]. Although factors determining cancer cell mobilization, such as Rho family small G proteins, have been extensively studied [3], the association between cell cycle regulation and cellular mobility of cancer cells remains unclear. To elucidate this dynamic interaction it would be valuable to observe the spatiotemporal properties of cell cycle regulation and cell mobility simultaneously *in vivo*.

Recently, intravital multiphoton microscopy was employed for dissecting intact cellular phenomena in various biological systems, such as the immune response [4], [5], inflammatory reactions [6], and bone remodeling [7]. This advanced imaging technique has enabled us to grasp the dynamic behaviors of living cells in tissues and organs. Cancer cells are also highly mobile and their migratory behaviors have been evaluated using this imaging technique [8]–[10], although its correlation with the proliferative nature of cells remains elusive.

Here, we succeeded in visualizing dynamic events during cancer cell invasion and metastasis by using intravital multiphoton microscopy. By means of fluorescent ubiquitination-based cell cycle indicator (Fucci), a special fluorescent protein probe used for monitoring the cell cycle in live cells, we identified a close association between the cell cycle and the mobilizing properties of cancer cells.

## Results

### Dynamic visualization detects cell cycle-associated cancer cell mobilization and invasion *in vivo*

**Figure 1:**
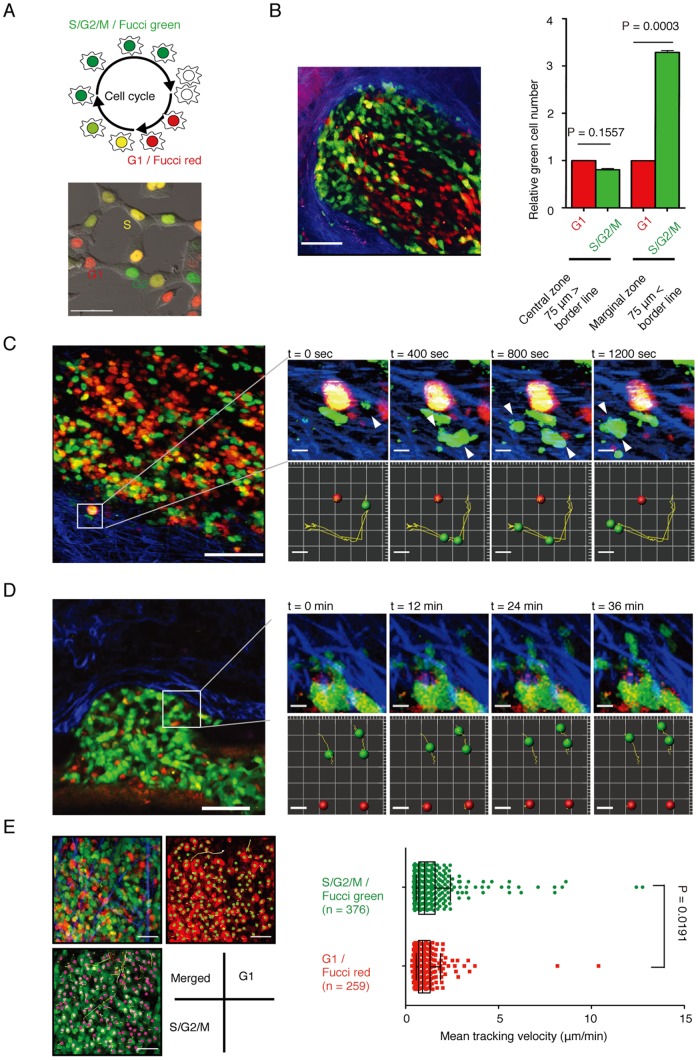
Visualization of cell cycle-dependent cancer cell mobilization and invasion. (A) Establishment and analyses of HCT116 colon cancer cells stably expressing Fucci. (*Upper*) The Fucci system enables monitoring of the cell cycle in live cells in real time. The nuclei of cells in the G1/G0, early S, and S/G2/M phases are labeled red, yellow, and green, respectively. (*Lower*) Snapshots of Fucci-expressing HCT116 cells. Scale bars represent 20 μm. (B) Intravital multiphoton imaging of Fucci-positive HCT116 cells inoculated into NOD/SCID mice. (*Left*) A representative image of Fucci-expressing HCT116 cells implanted in the cecum (green: Fucci-green (mAG), S/G2/M; red: Fucci-red (mKO2), G1; blue: collagen fibers (second harmonic generation (SHG) imaging)). Scale bars represent 75 μm. (*Right*) Quantification of the numbers of Fucci-green and -red HCT116 cells in different areas of inoculated tumors. Central and marginal zones were defined as areas further or closer than 75 μm from the border between the tumor and normal tissues, respectively. (C) Representative image at the edge of a Fucci-expressing HCT116 tumor mass. The entire area (*left*) and a time series (*right*) of magnified images (one per 400 s) of cancer cells invading the interstitium (green: Fucci-green (mAG), S/G2/M; red: Fucci-red (mKO2), G1; blue: collagen fibers (SHG imaging) (see also Movie S1). Actual images (*upper* panels) and cell trajectories (*lower* panels) are shown. Scale bars represent 100 μm (*left*) and 10 μm (*right*). (D) Representative image of extravasating Fucci-expressing HeLa cells. The entire area (*left*) and a time series (*right*) of magnified images of cancer cells extravasating from blood vessels (one per 12 min) (green: Fucci-green (mAG), S/G2/M; red: Fucci-red (mKO2), G1; blue: collagen fibers (SHG imaging) (see also Movie S2). Actual images (*upper* panels) and cell trajectories (*lower* panels) are shown. Scale bars represent 100 μm (*left*) and 10 μm (*right*). (E) Cellular motility in Fucci-green- and -red-positive cells was measured for 4 h (see also Movie S3). (*Left*) Green and red spheres represent Fucci-green- and -red-positive cells, respectively, and yellow lines show the associated trajectories. Scale bars represent 100 μm. (*Right*) Mean tracking velocities of Fucci-green- and -red-positive cells. Data (n = 379 for Fucci green and n = 259 for Fucci red) were obtained from individual cells in three independent experiments. The velocities of the two groups were compared by Mann-Whitney *U*-test (p = 0.0191). The median and interquartile ranges for each group are overlaid on the dot plots.

We utilized intravital multiphoton microscopy and Fucci technology [11] to study the cell cycle and migration in a living system. In this Fucci system, Geminin (GMNN), a nuclear protein enriched in the S/G2/M phases, and Cdt1, enriched in the G1 phase, were respectively marked with green- and red-fluorescent proteins (Figure 1A, *upper* panel). Fucci-expressing HCT116 human invasive colon cancer cells (Figure 1A, *lower* panel) were inoculated into the cecum or subcutaneous tissues of an immunocompromised NOD/SCID mouse [12]–[14]. Four weeks after implantation, tumors were observed intravitally. We preferentially detected S/G2/M-phase Fucci-green cells along the marginal areas of cancer invasion heads after inoculated into the cecum wall (Figure 1B). Similar distributional changes in Fucci-green and -red cells were detected when the cancer cells were inoculated into the mesentery or colon wall (Figure S1). The preferential distribution of cancer cells in the S/G2/M phases was also observed in surgically resected human colon cancer samples (Figure S2). Cancer cells at invasion heads were preferentially stained with antibodies against GMNN [15], [16] compared with those in non-tumor regions or the tumor centers.

Next, we examined the dynamic nature of cancer cells *in vivo*. Fucci-expressing HCT116 cancer cells were highly mobile upon inoculation into subcutaneous tissues, and some cancer cells were actively invading, appearing to ‘dive’ into the surrounding interstitium during imaging time-courses (Figure 1C; Movie S1). Notably, almost all of the diving cells were green (Figure 1C, *arrowheads*), suggesting that cancer cell motility and invasion might be dependent on the cell cycle. Moreover, we could detect their migratory movements during extravasation for metastasis (Figure 1D; Movie S2). Some cells were found to migrate out from cancer cell aggregates stuck within blood vessels, and these were also all green. A certain period of observation (for up to 2 h) of tumor central regions resulted in capture of basal sluggish behaviors, as well as streaming movement with blood flow, of various cancer cell types, which allowed us to image a sufficient number of cells for quantification (Figure 1D; Movie S3). Detailed statistical analyses of cell tracking velocity clearly demonstrated that green cancer cells (in the S/G2/M phase) have significantly higher motility than red G1 cells (mean tracking velocity 1.39±0.08 μm/min for Fucci-green vs. 1.09±0.06 μm/min for Fucci-red; p = 0.00191) (Figure 1E). We confirmed that a certain period of intravital imaging (up to 3 h) did not affect the mobility of cancer cells (Figure S3). By tracking individual cells over a period of time, we excluded the possibility that such mobility change in the S/G2/M phases reflected the motion regarding cytokinesis. These results indicate that certain molecules preferentially expressed in the S/G2/M Fucci-green phases facilitate the migration and invasion of cancer cells.

### Identification of ARHGAP11A as a cell cycle-dependent mobility-controlling molecule

**Figure 2:**
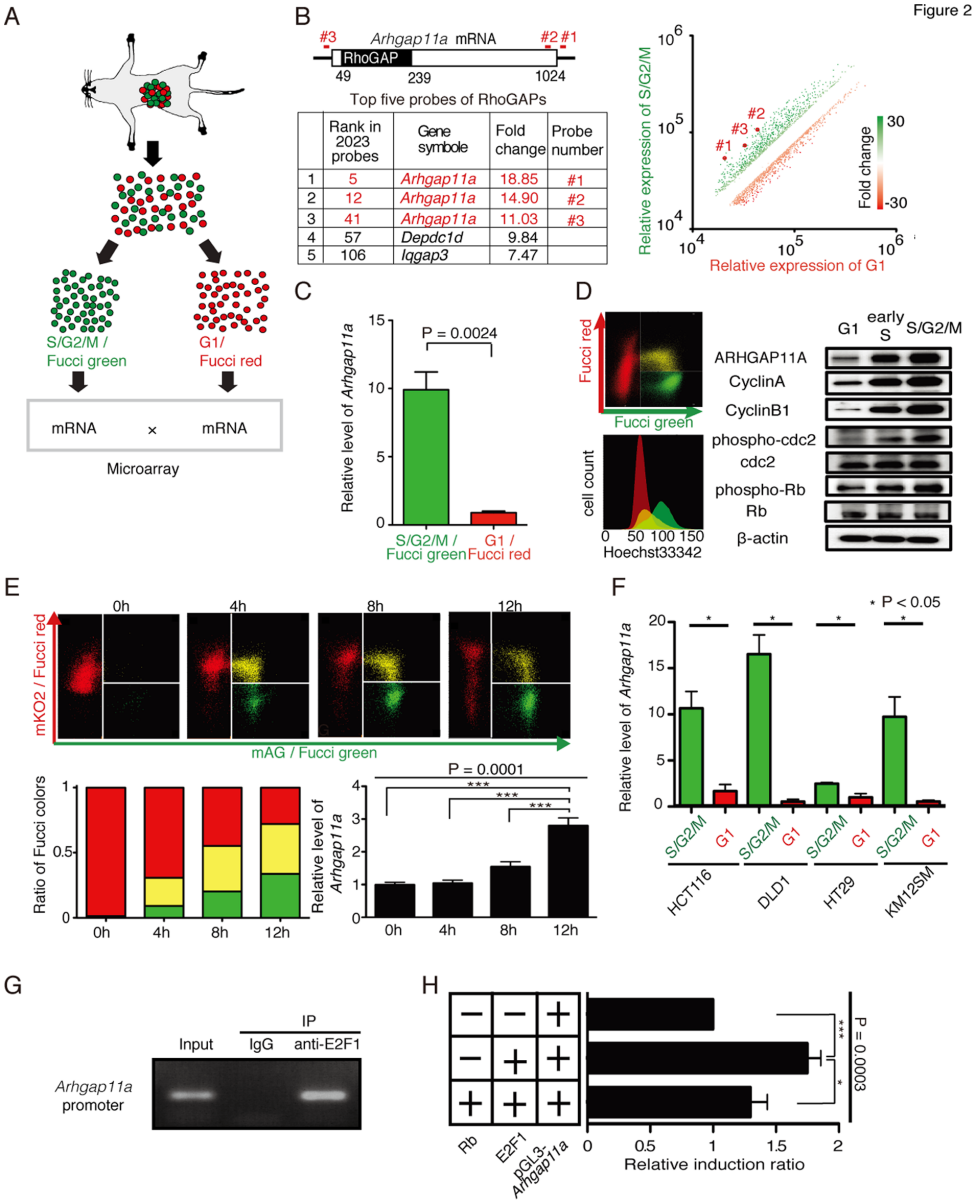
Identification of ARHGAP11A as a cell cycle-dependent mobility- controlling molecule. (A) Fucci signal-based microarray analyses. Fucci-positive HCT116 cells were separated into Fucci-green (mAG)- and Fucci-red (mKO2)- positive cells by FACS. mRNA was extracted from these cells and compared by microarray analysis (two dye-swap experiments, giving four independent microarray analyses). (B) In total, 2,023 probes (1,656 genes) showed >twofold changes in expression (Table S1) (P>0.05). Of them, *Arhgap11a* was highly ranked, and all three probes for *Arhgap11a* were among the top-ranked probes for RhoGAPs. The three probes were specific for the indicated potions of the *Arhgap11a* mRNA (*left*). The three marked dots (#1, #2, #3) in the scatter plots represent fold changes (*right*). (C) Cell cycle-dependent expression of *Arhgap11a* mRNA was confirmed by qPCR. The expression data were normalized to *Gapdh* (n = 3). (D) Cell cycle-dependent expression of *Arhgap11a* proteins. (*Right*) Flow cytometric analyses of Fucci-expressing HCT116 cells. Cell cycle profiles were color-coded: G1, red; early S, yellow; and S/G2/M, green (*upper right*). DNA contents were measured by Hoechst33342 fluorescence of (*lower right*), confirming that Fucci signals accurately represent cell cycle levels (n = 3). (*Left*) Cell cycle-dependent expression of ARHGAP11A and cell cycle markers, as determined by Western blotting (n = 3). (E) Time-dependent expression of *Arhgap11a* during progression of the cell cycle from G1 to S/G2/M. Fucci-red (mKO2)-positive HCT116 cells were sorted using a FACSAria cell sorter and were cultured for the indicated periods of time. Flow cytometry analyses (*upper*) and ratios of Fucci colors (*left*) are shown for each time point. (*Right*) Relative expression of *Arhgap11a* was examined by qPCR (n = 6). Data were analyzed by one-way ANOVA (*p* = 0.0001) and Bonferroni's multiple comparison test (*** *p*<0.01). (F) Cell cycle-dependent *Arhgap11a* expression in various human colon cancer cell lines. Fucci was introduced into different human colon cancer cell lines (HCT116, DLD1, HT29, and KM12SM). In all of the cell lines, *Arhgap11a* expression was significant higher in S/G2/M (*green*) than in G1 cells (*red*). (G) A chromatin immunoprecipitation (ChIP) assay with an anti-E2F1 antibody showed that E2F1 bound to the putative E2F-binding site in the *Arhgap11a* promoter (n = 3). (H) Luciferase reporter assay of the *Arhgap11a* promoter region (−500 bp), including the E2F-binding site (GTTTCGCGC) at −20 bp from the transcription starting point. Co-transfection with E2F1 enhanced transcriptional activity, whereas simultaneous expression of Rb blocked it. Values for luciferase activity were normalized across each experiment and, to control for differences in transfection efficiency, to β-galactosidase.

To elucidate the molecular basis of the control of cell cycle-dependent motility, we performed cDNA microarray-based comparative analyses among Fucci-green and -red cells cultured *in vivo* (Figure 2A). In the microarray analysis, 2,032 probes (1,656 genes) showed >two fold changes in expression (Figure 2B; Table S1). As anticipated, most of these genes encode proteins associated with cell division and mitosis. Based on gene ontology categories, we extracted genes related to cellular movement from the 1,656 candidates and found that an uncharacterized Rho GTPase-activating protein (RhoGAP), *Arhgap11a*, was preferentially expressed in green S/G2/M phase cancer cells (Figure 2B, Table S1). All of the three probes for *Arhgap11a* gene were highly ranked (5th, 12th, and 41st) among the 2,023 probes. It has been demonstrated that Rho family small G proteins such as Rho, Rac, and Cdc42, and their regulatory molecules, such as RhoGAP, cooperatively control cellular motility in both normal and cancer cells [17]–[21]. The preferential expression of *Arhgap11a* in Fucci-green cells was confirmed at both the mRNA (Figure 2C) and protein levels (Figure 2D). Additionally, we demonstrated a time-dependent gradual increase in *Arhgap11a* expression during progression through the cell cycle from G1 to S/G2/M (Figure 2E; Figure S4), strengthening the concept that *Arhgap11a* expression is controlled in a cell cycle progression-dependent manner. Cell cycle-dependent expression of Arhgap11a was also detected in other colon cancer cell lines besides HCT116, such as DLD1, HT29 and KM125M (Figure 2F) and HeLa (Figure S5) as well as in non-cancer cell lines such as HEK293 cells (Figure S6), suggesting the presence of a general mechanism for this characteristic expression regulation of *Arhgap11a* in many cell types. To elucidate the mechanism underlying the cell cycle-dependent expression of Arhgap11a, we further examined the transcriptional control of this gene. E2F family transcription factors have been documented to function in a cell cycle-dependent manner [1]. We noticed that a putative E2F-binding sequence (TTTCGCGC) [23] was located at −27 to −20 base pairs from the transcription initiation site of Arhgap11a. Chromatin immunoprecipitation (ChIP) experiments demonstrated the direct association of E2F1 with that region (Figure 2G), which may be involved in the cell cycle-dependent transcriptional activation of this locus. A luciferase reporter assay showed E2F1-dependent transcriptional activation of the Arhgap11a promoter, which was blocked by association with the Rb protein (Figure 2H), suggesting the possible role of E2F/Rb pathways in the transcriptional regulation of Arhgap11a. We fully realize the involvement of other transcriptional factors since Arhgap11a was substantially expressed also in G1 phase (Figure 2D), although we can assume Rb/E2F pathway would be at least responsible for the increase in Arhgap11a expression in S phase.

### ARHGAP11A is a GTPase-accelerating protein for Rho, but not for Rac or Cdc42, and inhibits Rho-dependent cellular phenomena

**Figure 3:**
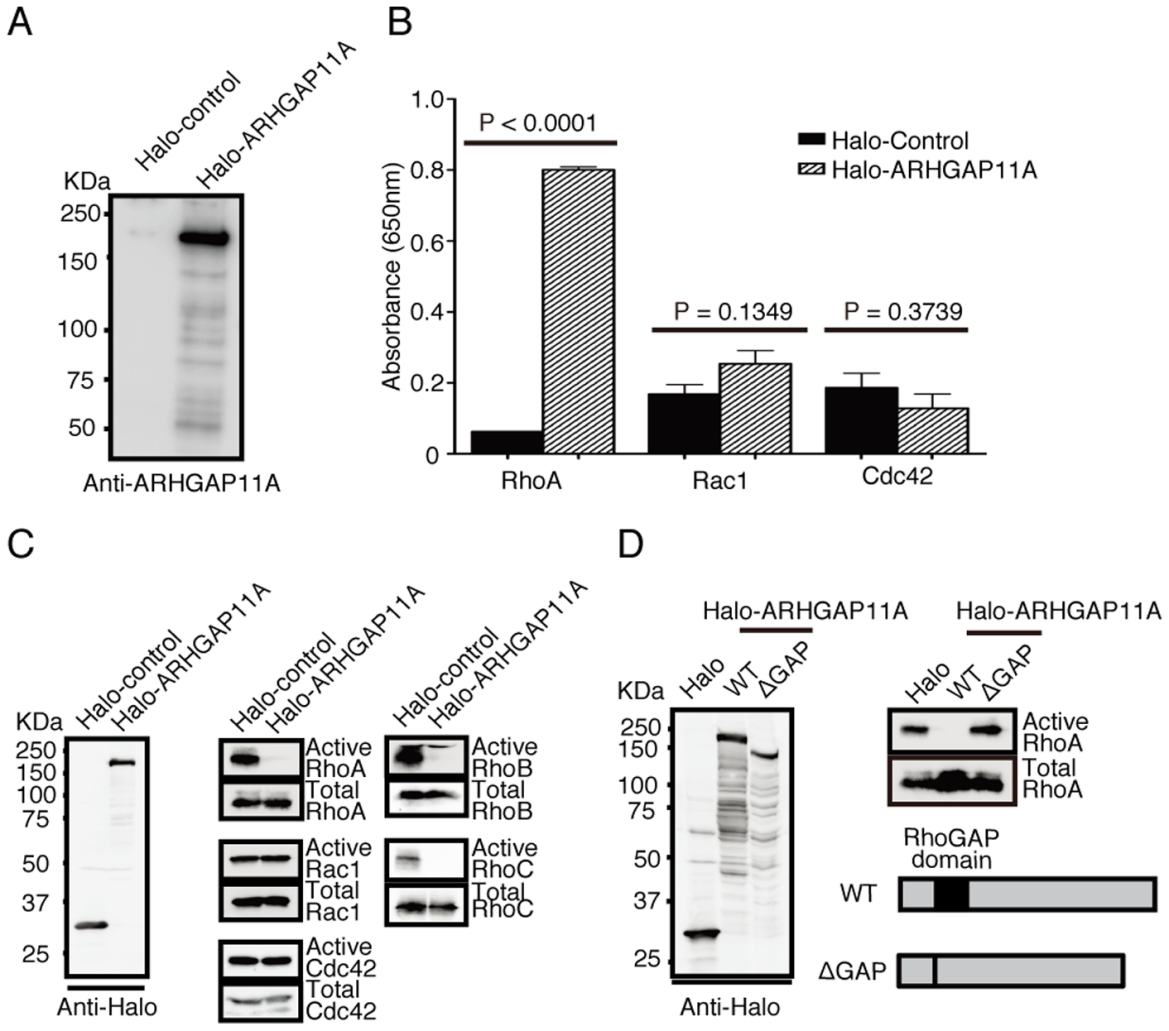
GTPase-activating properties of ARHGAP11A. (A) Halo-Tagged ARHGAP11A and Halo-Tag proteins expressed and purified from HEK293 cells. (B) Detection of GTPase activity by quantifying inorganic phosphate released by GTP hydrolysis by Rho family proteins. ARHGAP11A enhanced the GTPase activity of RhoA, but not of Rac1 or Cdc42. (C) Detection of active and total forms of various Rho-family proteins (RhoA, RhoB, RhoC, Rac1, and Cdc42) in HEK293 cells transfected with Halo-ARHGAP11A or its control. Expression of ARHGAP11A reduced the amounts of the active forms of RhoA, RhoB, and RhoC. (D) Assessment of mutant ARHGAP11A without the putative GAP domain (ΔGAP).

ARHGAP11A had already been cloned and listed in an NCBI database, but its molecular function had not yet been characterized. Notably, it was unclear whether its putative GAP domain indeed exerts a GTPase-accelerating effect. To determine its function, we isolated Halo-tagged ARHGAP11A (or Halo-Tag only as a control) expressed in HEK293 cells (Figure 3A), and incubated it *in vitro* with several Rho family proteins, such as RhoA, Rac1, and Cdc42 (Figure 3B). In this assay, we measured GAP activity by detecting inorganic phosphate released due to GTP hydrolysis of Rho proteins [24]. This cell-free in vitro assay showed that ARHGAP11A greatly accelerated GTP hydrolysis of RhoA, but not that of Rac1 or Cdc42 (Figure 3B). The amounts of active forms of Rho family proteins were also assessed by pull-down assay with GST-Rhotekin (for Rho) or GST-CRIB (for Rac and Cdc42) in HEK293 cells [25]. Transfection of ARHGAP11A reduced the amounts of active RhoA, RhoB, and RhoC, but not of Rac1 or Cdc42 (Figure 3C). We also confirmed that this RhoGAP activity was essentially abolished when its putative GAP domain was deleted (Figure 3D). These results clearly confirm that ARHGAP11A is a GAP specific for Rho, but not for Rac or Cdc42, whose effect is mediated by its predicted GAP domain.

**Figure 4:**
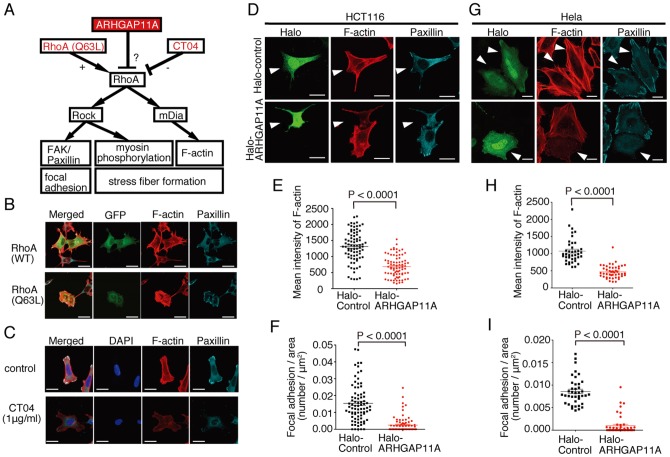
Functional analyses of ARHGAP11A in RhoA-mediated cellular reactions in HCT116 colon cancer cells. (A) Schematic illustration of RhoA-mediated cellular reactions. (B) Effect of overexpression of wild-type (WT) or constitutively active (Q63L) RhoA on the formation of F-actin stress fibers (visualized using Alexa 568-phalloidin) and focal adhesions (stained with anti-paxillin). GFP was co-transfected to identify the transfected cells. Scale bars represent 15 μm. (C) Effect of CT04, a potent RhoA inhibitor, on the formation of F-actin stress fibers (visualized using rhodamine-phalloidin) and focal adhesions (stained with anti-paxillin). Nuclei were stained with DAPI. Scale bars represent 15 μm. (D) Effect of overexpression of Halo-Tagged ARHGAP11A or its control on the formation of F-actin stress fibers (visualized using Alexa 568-phalloidin) and focal adhesions (stained with anti-paxillin) in HCT116 cells. Arrowheads identify Halo-Tag-expressing cells (labeled with Oregon green-conjugated Halo-Tag ligand). Scale bars represent 10 μm. (E) Quantification of mean intensities of F-actin in Halo-control (n = 80) and Halo-ARHGAP11A-expressing (n = 80) HCT116 cells. Data were compiled from three independent experiments. (F) Quantification of focal adhesions in Halo-control (n = 80) and Halo-ARHGAP11A-expressing (n = 80) HCT116 cells. Data were compiled from three independent experiments. (G) Effect of overexpression of Halo-Tagged ARHGAP11A or its control on the formation of F-actin stress fibers (visualized using Alexa 568-phalloidin) and focal adhesions (stained with anti-paxillin) in HeLa cells. Arrowheads identify Halo-Tag-expressing cells (labeled with Oregon green-conjugated Halo-Tag ligand). Scale bars represent 10 μm. (H) Quantification of mean intensities of F-actin in Halo-control (n = 80) and Halo-ARHGAP11A-expressing (n = 80) HeLa cells. Data were compiled from three independent experiments. (I) Quantification of the number of focal adhesions in Halo-control (n = 40) and Halo-ARHGAP11A-expressing (n = 46) HeLa cells. Data were compiled from three independent experiments.

Active RhoA has been shown to stimulate focal adhesion and stress fiber formation by the activation of Rho-associated protein kinase (ROCK) and/or mDia (Figure 4A) [26]. Concordantly, exogenous expression of constitutively active RhoA (RhoA-Q63L) [27] in HCT116 cells induced aberrant increases in F-actin stress fiber and focal adhesion formation, visualized by means of paxillin aggregates (Figure 4B). In contrast, suppression of Rho activity by CT04 (1 μg/ml), a potent Rho inhibitor [28], reduced the formation of stress fibers and focal adhesions (Figure 4C). Under these experimental conditions, additional expression of ARHGAP11A significantly inhibited the formation of both F-actin stress fibers (Figure 4D, *middle* panel, and 4E) and focal adhesions (Figure 4D, *right* panel, and 4F). Expression of ARHGAP11A also reduced the level of phosphorylated myosin light chain (pMLC) (Figure S7). In summary, ARHGAP11A, as a RhoGAP, suppressed Rho-dependent phenomena, such as focal adhesion and stress fiber formation, in HCT116 cancer cells. We also confirmed the function of ARHGAP11A in HeLa cells (Figure 4G–I).

### ARHGAP11A stimulates cancer cell motility by enhancing Rac activity

**Figure 5:**
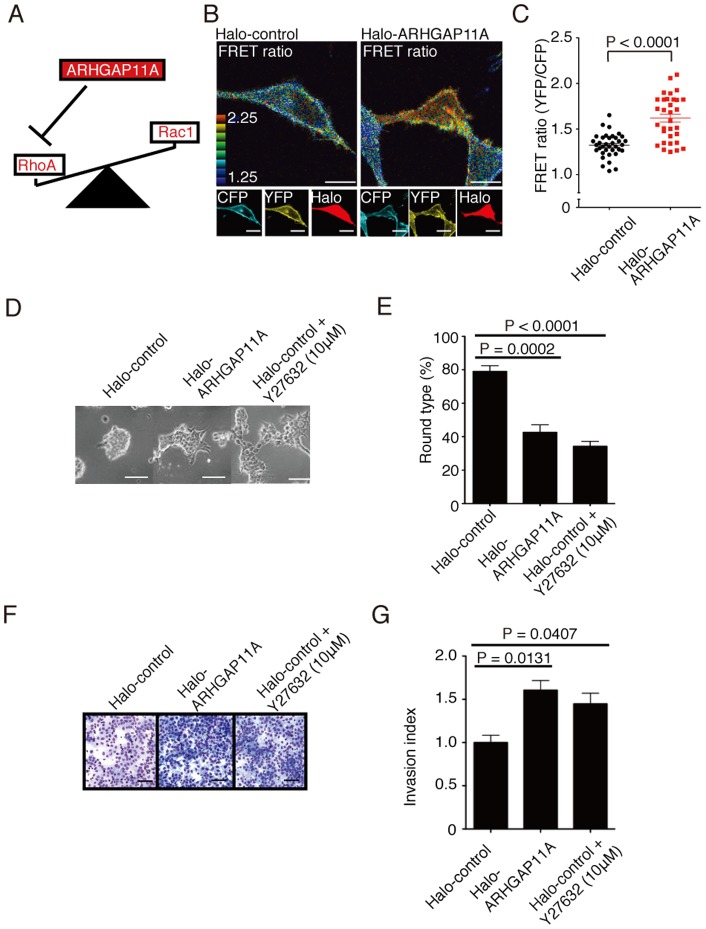
Relative augmentation of Rac1 activity and increased invasive migration in ARHGAP11A-expressing cancer cells. (A) Schema representing the balance between RhoA and Rac1 for cell migration. (B) Analyses of Rac1 activity at the single-cell level in HCT116 cells expressing Halo-ARHGAP11A or its Halo control. Representative images of Raichu-Rac1-expressing HCT116 cells under Halo-control (*left*) or Halo-ARHGAP11A transfection (*right*) conditions. Rac1 activity was monitored by CFP/YFP FRET ratios derived from Raichu-Rac1. Expression of Halo-Tag was identified with TMR-conjugated Halo-Tag ligand. The scale bar represents 5 μm. (C) Quantification of FRET ratios in Halo-control (n = 30) and Halo-ARHGAP11A-expressing (n = 30) HCT116 cells. (D) Three-dimensional culture of HCT116 transfected with Halo-control or Halo-tagged ARHGAP11A, supplemented with Y27632 (for Halo-control only). The scale bar represents 50 μm. (E) Proportions of round-type HCT116 in 3D culture transfected with Halo-ARHGAP11A or Halo-control. Round-type cells were counted in three visual fields for each of three independent experiments. Columns represent the mean ± s.e.m. (F) *In vitro* invasion assay using 3D Matrigel plate. Migrated cells were visualized by staining culture membrane with Diff Quik stain (Dade Behring). HCT116 transfected with Halo-ARHGAP11A or Halo-control, and wild-type HCT116 treated with Y27632 were used in the assay. (G) Quantification of invasion indices from 3D Matrigel plate assays. Invasion indexes were calculated according to the equation shown in the Method section. Columns represent the means ± s.e.m.

Cell motility is known to be reciprocally regulated by diverse Rho family small G proteins [29]. Whereas active RhoA (or Rho or RhoC) stabilizes cytoskeletons by enhancing stress fiber and focal adhesion formation, activation of Rac1 (or Cdc42) makes cells flexible and mobile, leading to the formation of lamellipodia or filopodia [29]. The activities of the counteracting proteins RhoA and Rac1 are mutually controlled [30], and inhibition of one results in the relative augmentation of the other (Figure 5A). Here, by using Raichu-Rac1, a FRET-based biosensor of Rac1 activity at the single-cell level [31], [32], we examined Rac1 activity in HCT116 cells. Rac1 activity was significantly higher in ARHGAP11A-expressing cells compared to mock-transfected (transfected with Halo-Tag only) or non-transfected cells (Figure 5 B and C), suggesting the mechanism by which the suppression of Rho activity leads to counter-activation of Rac1 at the single-cell level. Similar ARHGAP11A-mediated activation of Rac1 was also observed in HeLa cells (Figure S8).

Next, we examined the effect of Rho inhibition by ARHGAP11A on the control of cancer cell morphology and mobility. To analyze the morphological properties of cancer cells, we a utilized three-dimensional (3D) Matrigel culture system (Figure 5 D and E) [33]. In results, over-expression of ARHGAP11A led to spindle-like shapes of HCT116 cells, representing an invasion-prone phenotype. Similar morphogenic changes could also be observed when Rho-mediated signaling was inhibited by Y27632, a potent ROCK inhibitor [34]. We further measured the *in vitro* migratory properties of HCT116 cell in 3D Matrigel plates (Figure 5 F and G) [35], and concordantly, overexpression of ARHGAP11A or Y27632 treatment enhanced migration of HCT116 *in vitro*. On the other hand, strong inhibition of Rho activity by high concentration of Y72632 blocked the migration (data not shown). These results clearly suggest that adequate level of RhoA inhibition such as achieved by overexpression of ARHGAP11A enhanced migratory activity of HCT116 cancer cells.

### Functional impact of ARHGAP11A on the mobilization of HCT116 human colon cancer cells *in vivo* and possibility of ARHGAP11A as a therapeutic target in invasive cancers

**Figure 6:**
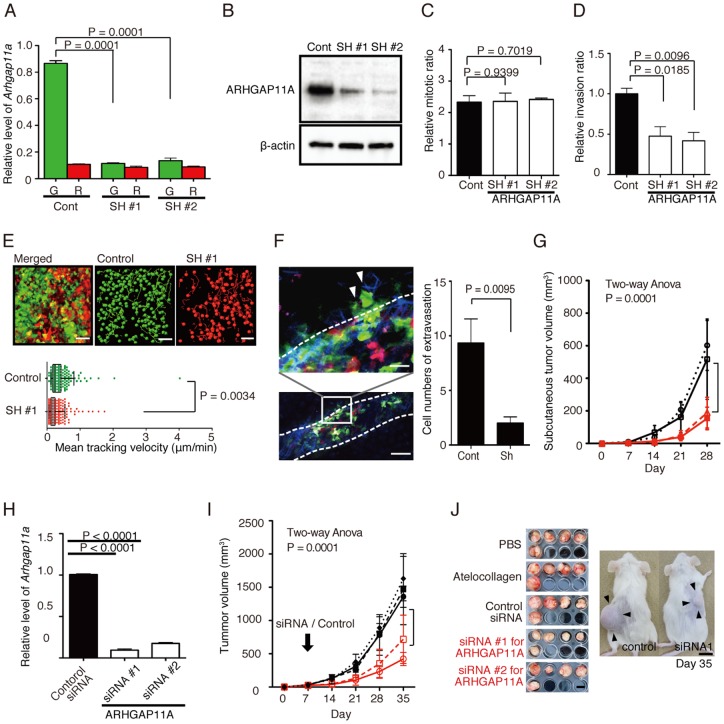
Functional analyses of ARHGAP11A-knockdown HCT116 human colon cancer cells. (A) Establishment of ARHGAP11A-knockdown HCT116 cell lines (SH #1, #2). Decreased ARHGAP11A mRNA expression was confirmed by qPCR. (B) ARHGAP11A protein expression in control and sh-knockdown HCT116 cells was assessed by Western blotting. (C) BrdU proliferation assay of these HCT116 cell lines. Columns represent the means ± s.e.m. (D) *In vitro* invasion assay using 3D Matrigel culture plates. Columns represent the means ± s.e.m. (E) *In vivo* functional analyses of ARHGAP11A-knockdown HCT116 cells. Representative images of control (*green*) and ARHGAP-knockdown (*red*) HCT116 cells inoculated subcutaneously into NOD/SCID mice (*upper*). A raw ‘merged’ image and images extracted from the green and red channels are shown. Cell motility was measured for 7 h. Green and red circles represent control and ARHGAP11A-knockdown SH#1 HCT116 cells, respectively, and yellow lines show their trajectories (see also Movie S4). The scale bar represents 50 μm. (*Lower*) Mean tracking velocities of control and SH cells. Data (n = 440 for the control and n = 215 for SH#1) were obtained from individual cells in two independent experiments. The velocities of the two groups were compared by Mann-Whitney *U*-test (p = 0.0034). The median and interquartile ranges for each group are overlaid on the dot plots. (F) Extravasation of control (*green*) and SH#1 (*red*) HCT116 cells. (*Left*) Green cells were preferentially detected in extravascular spaces, suggesting a high potency for extravasation. (*Right*) Average numbers of extravasated cells per visual field. Data were extracted from 10 visual fields. (G) In vivo tumor expansion of HCT116 cells. Wild-type control HCT116 cells (*black* circles) and HCT116 cells treated with scrambled control shRNA (*black* squares), SH#1 (*red* circles), and SH#2 (*red* squares) are shown. Cancer cells (1.0×10^6^/100 μl of PBS) were primarily inoculated into subcutaneous tissue. Tumor sizes were measured every week for 4 weeks after inoculation. Data represent the means ± s.e.m. of five independent experiments. Data were analyzed by two-way ANOVA (*p* = 0.0037). (H) Decreased expression of ARHGAP11A in HCT116 cells treated with siRNAs targeting ARHGAP11A (assessed by qPCR). (I) *In vivo* siRNA treatment of HCT116 tumors. Control HCT116 cells (5.0×10^6^) were implanted into NOD/SCID mice, and 1 week later were treated with PBS (*black filled* circles), atelocollagen (*black filled* triangles), scrambled control siRNA plus atelocollagen (*black filled* squares), or two siRNAs (#1 and #2) against ARHGAP11A plus atelocollagen (*red open* circles and squares, respectively). Tumor sizes were measured weekly. Data represent the means ± s.e.m. of five independent experiments. Data were analyzed by two-way ANOVA (*p* = 0.0001). (J) Images of tumors excised on day 35 (*left*). The scale bar represents 10 mm. (*Right*) A representative images of SCID mice bearing HCT116 human colon cancer cells, 35 days after treatment with an siRNA (siRNA#1) or with a scrambled RNA duplex (control), together with atelocollagen. The scale bar represents 10 mm.

To analyze the function of ARHGAP11A, we generated HCT116 cell lines in which ARHGAP11A expression was stably reduced by shRNA (SH). Reduced expression of ARHGAP11A was confirmed at both the mRNA (Figure 6A) and protein (Figure 6B) levels. A BrdU proliferation assay showed no differences between control and SH cells, suggesting that ARHGAP11A does not influence cell-intrinsic proliferation *in vitro* (Figure 6C). On the other hand, an *in vitro* cell invasion assay with a Matrigel plate showed that invasion ability was significantly reduced in SH cells (Figure 6D). Next we examined the role of ARHGAP11A in *in vivo* motility of inoculated cancer cells. By using intravital multiphoton imaging techniques, we showed that SH cells were less motile than control cells in subcutaneously inoculated tumors, clearly demonstrating that ARHGAP11A regulates the motility of HCT116 cancer cells in vivo (Figure 6E, Movie S4). We also found that SH cells were less able to migrate out from blood vessels than were control cells during extravasation of blood-resident cancer cells (Figure 6F). Finally, we investigated the role of ARHGAP11A in tumor expansion *in vivo* (Figure 6G). ARHGAP11A-knockdown SH cells exhibited less progression at day 28 compared with wild-type cells (SH#1, 6.47±0.33 mm; SH#2, 6.66±0.32 mm; wild-type, 9.76±0.82 mm; and SH control, 9.38±0.97 mm).

Next, we evaluated the potential of ARHGAP11A as a novel therapeutic target for inhibition of cancer progression. Subcutaneously implanted tumors were subjected to local injection of a siRNA targeting *Arhgap11a* or a scrambled control siRNA (Figure 6H). siRNAs were conjugated to atelocollagen to facilitate their introduction into target cells [36], [37]. We confirmed that siRNA injected *in vivo* was incorporated into inoculated cancer cells (Figure S9), although we could not exclude the possibility that siRNAs against ARHGAP11A may be taken up by and act on the stromal cells surrounding the tumors. *In vivo* siRNA treatment significantly reduced tumor expansion (1471±238.5 mm for scrambled siRNA, 422.1±44.5 mm for siRNA#1, and 717.6±162.7 mm for siRNA#2 at day 28), suggesting that ARHGAP11A is a promising therapeutic target for the treatment of invasive tumors (Figure 6J). Blockade of *Arhgap11a* would therefore be a conceptually novel anti-cancer therapy that would be expected to inhibit cancer cell mobilization and invasion into surrounding tissues.

### Enhanced expression of ARHGAP11A in surgically resected human colorectal cancers

**Figure 7:**
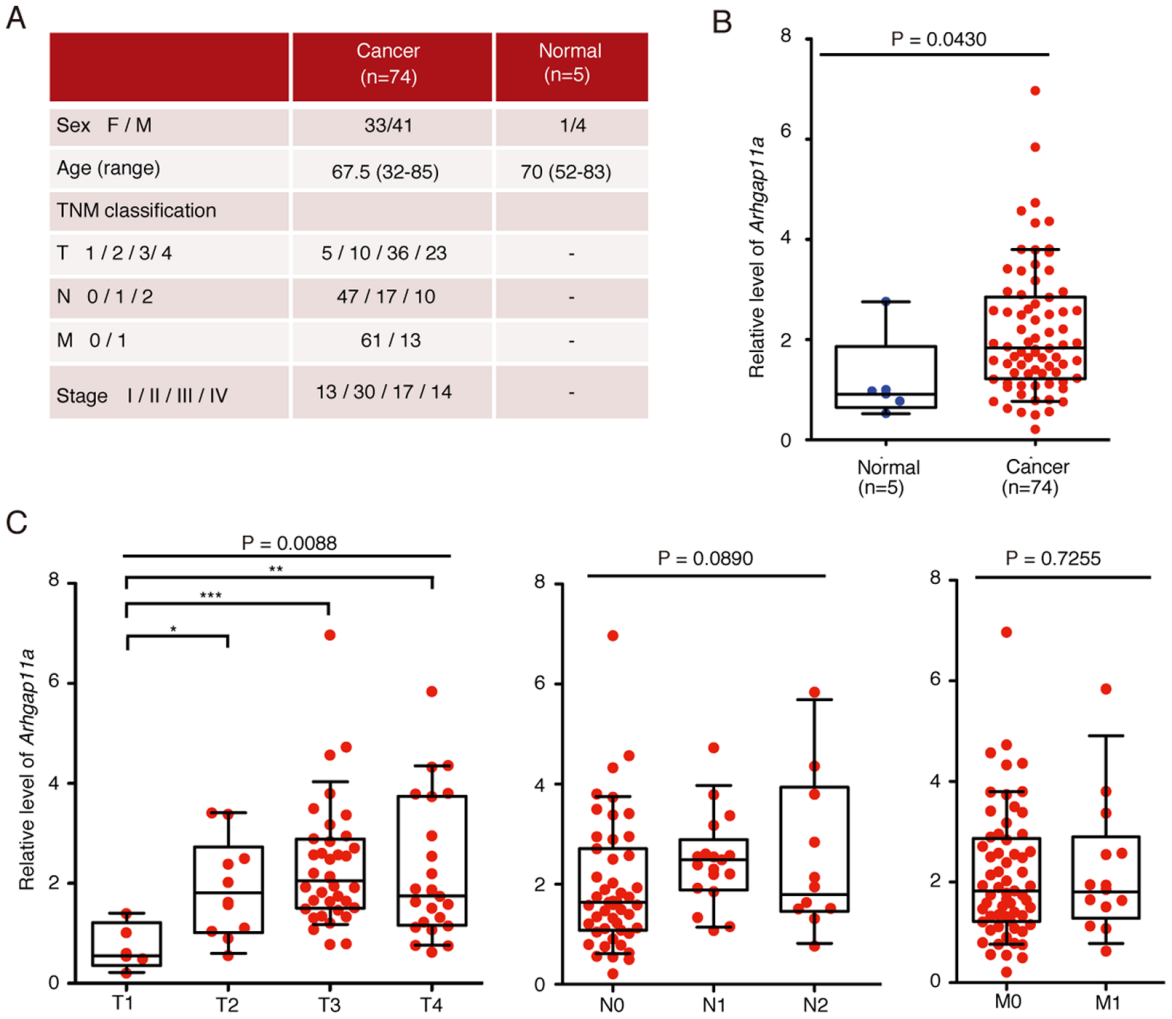
Enhanced expression of ARHGAP11A in surgically resected human colorectal cancers. (A) Clinicopathological data for human colon cancer patients (B) Microarray analysis of total RNA isolated from five normal tissue samples and 74 colorectal cancer specimens. The data points represent values from individual patients. The median and interquartile ranges are overlaid on each column. Relative expression of ARHGAP11A in normal colon tissues and colon cancer specimens (*p* = 0.0430 [Mann-Whitney *U*-test]). (C) Comparison of expression data based on TNM factors. There were significant increases in expression between T1 (tumor invades the submucosa) and T3 (tumor invades and penetrates the muscularis propria into the subserosa or non-peritonealized pericolic or perirectal tissue), and between T1 and T4 (tumor directly invades other organs or structures and/or perforates the visceral peritoneum). Data were analyzed by Kruskal–Wallis test and Dunnett's test (*** *p*<0.01, ** *p*<0.05, * *p*<0.1). The median and interquartile ranges for each group are overlaid on the dot plots.

Next, we investigated the clinical relevance of ARHGAP11A in human colon cancers *in situ*. Histological analyses of surgically dissected human colon cancer samples showed the preferential expression of ARHGAP11A at invasion sites (Figure S10), an expression pattern resembling that of GMNN (Figure S2). We also analyzed the expression of ARHGAP11A in clinical samples, including 74 colorectal cancers and five non-cancer mucosal tissue samples, using cDNA microarrays [35]. Expression of key molecules was assessed by qPCR, the results of which were reasonably correlated with the microarray data (Figure S11). To ensure the specificity of the analyses, target regions (cancers or normal tissues) were collected by laser capture microdissection [38]. Relative expression of ARHGAP11A was significantly higher in tumors than in normal tissues (Figure 7B). Comparison with data on clinical disease stage (UICC-TNM; Union International Centre le Cancer-Tumor Node Metastasis) [39] demonstrated a stepwise increase in ARHGAP11A expression during the progression of T factors from T1 (tumors limited to the submucosa) to T3 (tumors penetrate the propria muscularis and reach the outermost layers) (Figure 7C). These results strongly suggest that up-regulation of ARHGAP11A would be conducive to increased mobility and invasiveness of human colon cancer cells in patients.

We re-analyzed microarray data deposited in the NCBI Gene Expression Omnibus (GEO) database using the NextBio data mining framework [40] and found that ARHGAP11A was up-regulated in colorectal cancer tissues in eight of ten datasets (Figure S12) [41]. Moreover, ARHGAP11A was up-regulated in various cancer tissues other than colorectal cancers, including glioblastoma, lung cancer, breast cancer, gastric cancer, hepatocellular carcinoma and pancreatic cancers (Figure S13). In summary, ARHGAP11A, a cell cycle-dependent, mobility-controlling RhoGAP, is one of the most remarkable RhoGAP proteins in that it is associated with diverse epithelial cancer cell lines.

## Discussion

The results of this study present several significant aspects. First of all, it is surprising that cell mobility and thus cancer invasiveness are critically linked to the cell cycle via the action of ARHGAP11A. We now know that the cell cycle is not simply a cycle of cell division but also the basic machinery for regulation of diverse cellular events such as mobilization. Notably, we identified ARHGAP11A as a regulator of cell cycle-dependent motility. We found that expression of ARHGAP11A is altered in a cell cycle-dependent manner and *ARHGAP11A* may be one of the target genes of E2F1 which activates transcription in cell cycle-related fashion. On the other hand, we do not currently know the mechanisms by which ARHGAP11A is up-regulated in cancers. Nevertheless, it has been known that *Rb*, a tumor suppressor factor, is functionally inactivated in various cancers, leading to aberrant activity of E2Fs [22], which may result in enhanced expression of ARHGAP11A, although we cannot exclude the possibility that there are several other unidentified mechanisms to be involved. Additionally, we found that ARHGAP11A was modestly expressed in highly proliferative regions of normal mucosal tissues (Figure S6). We believe that this is a novel concept, not only in cancer biology but also in the broader research field of cell biology.

Recently, several reports have demonstrated possible direct connection between various canonical mediators for cell cycle progression and cellular mobility [42]–[44]. For instance, p27, a cyclin-dependent kinase inhibitor (CKI), was shown to interfere with Rho guanine nucleotide exchange factor (GEF) by binding to RhoA [43], while p21, another CKI, inhibits a Rho kinase, ROCK [44], [42]. On the other hand, RhoA was also reported to be associated with G1-S phase through transcriptional regulation of CKIs and cyclin D [45], although these were not fully demonstrated with their molecular mechanism and physiological significance in vivo The present study, presenting ARHGAP11A as a cell cycle-dependent mobility-controlling molecule, exhibits a comprehensive example underlying the critical coupling between cell proliferation and migration.

Cell cycle-dependent expression control of ARHGAP11A and the resultant cell cycle-dependent mobility changes were less prominent when cells were cultured *in vitro* (unpublished observation), suggesting the particular significance of this regulatory mechanism for cancer cell expansion in *in vivo* environments. In contrast, one previous report showed that basal motility of cells was reduced in the late G2 phase compared to G1 [46], which likely reflects preparation for cytokinesis, although the motility data were recorded *in vitro* and are not directly comparable to our *in vivo* analyses of cell motility.

A recent report showed that ARHGAP11A expression is increased and translocated to nucleus upon DNA damage in human glioblastoma [47]. And then, ARHGAP11A induces cell cycle arrest and apoptosis by binding and activating p53. The author indicated that some other ARHGAPs also control transcriptional activity of p53. Therefore, they consider that down-regulation of ARHGAPs in cancer associates with malignant phenotypes. However, we revealed that ARHGAP11A was up-regulated in various cancers including glioblastoma, whereas almost ARHGAP isoforms were down-regulated. In fact, we showed that ARHGAP11A promotes migration and invasion of cancer cells, by inhibition RhoA and resultant counter-activation of Rac1.

Regulation of cancer cell mobility by Rho family proteins has been investigated extensively. There is abundant published evidence showing the critical role of Rho signaling, including that mediated by ROCK, on cancer cell invasion and metastasis [26], [48]. We also showed that strong inhibition of RhoA signaling rendered cells deformed and inhibited mobilization, although excessive activation of RhoA signaling (such as by RhoA-Q63L) signaling by expression of constitutive active RhoA (Q63L) made the cells too rigid (Figure 4B). In this study, we showed that adequate inhibition of Rho activity, by the expression of ARHGAP11A, a RhoGAP specific for RhoA, enhanced mobilization, suggesting the significance of ‘fine tuning’ of Rho activation tone. Furthermore, ARHGAP11A induced mobilization by counter-activation of Rac1, which is concordant with previous reports showing that the inhibition of Rho signaling led to morphological changes and mobilization via activation of Rac1 [49]. ARHGAP11A-high Fucci-green cells seemed to exhibit streaming-like migration with elongated shapes during extravasation, which may also be consistent with a previous report that elongated movement is mediated mainly by Rac1 [21], [50].

Many researchers have been so far extensively studying on novel cancer treatments targeting invasion and/or metastasis. Focal adhesion kinase (FAK) has been fully investigated as a promising target because it is highly expressed in metastatic cancers and has been shown to be involved in their various features including tumor growth, invasion, metastasis and angiogenesis [51]. In fact, FAK kinase inhibitors had already been tried clinically, although these approaches was halted because of their off target effects derived from multiple consensus sequences in the kinase domain. In addition, a recent report shows that intermediate decrease in FAK expression (with FAK-heterozygous allele), or incomplete inhibition by a low dose of pharmacological FAK inhibitor unexpectedly enhanced angiogenesis and tumor growth [52]. Matrix metalloproteinases (MMPs), a family of zinc-dependent endopeptidases, are another potential therapeutic target to invasion/metastasis, and also epithelial-mesenchymal transition (EMT) [53]. MMP inhibitors have been rapidly developed for therapeutic use, although the clinical trials have been withdrawn. This was because each MMPs have distinct, sometimes opposite, effects on tumor angiogenesis, and lack of isoform specific inhibitors rather increased the occurrence of malignancy. Here, we propose that ARHGAP11A may be a good novel candidate as a target against invasive cancers, namely blocking cancer cell progression by inhibiting invasive properties. Because of its oncostatic nature, this therapy may not be used alone. Nevertheless, we detected increased expression of ARHGAP11A in highly invasive tumor (Figure S13){Kupershmidt, 2010 #428}, and this therapy would therefore be useful for treating highly invasive cancers, chief among them glioblastoma and pancreatic cancer, for which there are currently limited therapeutic approaches. Furthermore, ARHGAP11A is specifically increased in a wide array of cancers, compared as other ARHGAP family members (Figure S12), suggesting the possibility that we could develop therapeutically effective inhibitors with less adverse effects. Consequently, ARHGAP11A, a RhoGAP preferentially expressed in invasive human cancers, may become a promising target for cancer therapy.

## Materials and Methods

### Ethics Statement

All animal experiments were performed with the approval of the Animal Experiments Committee of Osaka University (permission number: IFReC-AP-H21-01-7). All surgery was performed under isoflurane anesthesia, and all efforts were made to minimize suffering. Clinical tissue samples for analyses were obtained with written informed consent, under the protocol approved by the Osaka University Research Ethic Committee.

### Quantitative real-time RT-PCR and protein analysis

Quantitative real-time PCR, preparation of cell lysates, and Western blotting were performed according to standard protocols. The primers used are listed in Table S2. The antibodies used for Western blotting are listed in Table S3.

### Lentiviral transfection and stable cell sorting

EGFP (pEGFP-C1, Clontech), DsRed2 (pDsRed2-C1, Clontech), mAG-hGem(1/110) and mKO2-hCdt1(30/120) (provided by Dr. Miyawaki, RIKEN-BSI, Japan) were cloned into the CSII-EF-MCS vector (provided by Dr. Miyoshi, RIKEN-BRC, Japan) and transfected into HEK293T cells with packaging plasmids [54]. Stable transformants were selected using a FACSAria cell sorter (BD Biosciences). EGFP and Fucci-green (mAG), and DsRed2 and Fucci-red (mKO2), were excited by 488-nm laser lines, and their emission was detected with 530/30BP and 585/42BP filters, respectively.

### Establishment of knockdown cell lines

MISSION shRNA plasmids for human ARHGAP11A (Table S4), a scrambled control shRNA, and lentiviral packaging mix were purchased from Sigma. Lentivirus-mediated transfection of shRNAs was performed according to the manufacturer's instructions.

### Chromatin immunoprecipitation (ChIP) analysis

ChIP was performed essentially as described previously [55] with an anti-human E2F1 polyclonal antibody (sc-251x, Santa Cruz) or a control IgG (H-270, Santa Cruz). The primers used are listed in Table S5.

### Purification of ARHGAP11A

Purification of the Halo-tag control and ARHGAP11A were performed according to the manufacturer's instructions. Briefly, HEK293 cells were transfected with control and ARHGAP11A Halo-tag expression vectors. Two days after transfection, transfected cells were collected and lysed with a lysis buffer. The resulting lysates were added to HaloLink Resin (Promega) and incubated for 6 h at 4°C with constant mixing. The resin was washed three times, and Halo-ARHGAP11A was cleaved by HaloTEV protease (Promega). After centrifugation, the supernatants were collected. Purified proteins were validated by Western blotting using an anti-ARHGAP11A antibody (Table S3).

### *In vitro* RhoGAP assay

A RhoGAP assay (Cytoskeleton) was performed according to the manufacturer's instructions. Briefly, 9 μl of purified ARHGAP11A (0.68 μg/μl) was added to reaction buffer and 5 μl of a small GTPase protein (RhoA, Rac1 or Cdc42, 0.78 μg/μl) and incubated with 10 μl of 800 μM for 20 min at 37°C. Then, 120 μl of CytoPhos reagent (Cytoskeleton) was added for 10 min to determine the amount of phosphate generated by the hydrolysis of GTP. The absorbance of each sample at 650 nm was read.

### Pull-down activity assays of Rho, Rac, and Cdc42

Activities of Rho, Rac, and Cdc42 were measured using glutathione-S-transferase (GST)-CRIB and GST-Rhotekin, as described previously [25]. For assays, 24 h after transfection of HEK293 cells with Halo-ARHGAP11A or Halo-control vector (100-mm-diameter dish), the cells were lysed in 400 μl of a lysis buffer containing 15 μg of GST-CRIB (for Rac and Cdc42) or GST-Rhotekin (for Rho assays). The lysates were centrifuged at 20,000×g for 10 min, and the resulting supernatants were then incubated with glutathione-Sepharose (20 μl) for 1 h at 4°C. Glutathione–Sepharose was precipitated by centrifugation, and the bound proteins were probed with anti-Rac1, anti-Cdc42, anti-RhoA, anti-RhoB, and anti-RhoC antibodies.

### Immunocytochemistry

Cells were grown in incubation chambers coated with BioCoat collagen I (BD Biosciences). When necessary, Halo-tag was labeled with Halo-tag Oregon green ligand (Promega) according to the manufacturer's protocol. Cells were fixed for 10 min at room temperature in PBS containing 4% (w/v) paraformaldehyde and were then permeabilized in PBS containing 0.5% (w/v) Triton X-100 and 2 mg/ml BSA for 10 min. The cells were incubated with primary antibodies, and then labeled with secondary antibodies. All images were taken with an A1 confocal microscope (Nikon). For quantification of paxillin number and F-actin mean intensity, transfected cells were imaged for each condition. Images of transfected cells were acquired using the same parameters, without automatic scaling and gain adjustment, to avoiding pixel saturation. In all cases, the mean F-actin fluorescence intensity over the whole cell area was measured using the commercially available software NIS-Elements (Nikon) (Figure S14). Paxillin numbers were counted using an analysis tool, “*Object counter*”, in NIS-Elements

### FRET imaging of Rac1 activity

FRET images were obtained and processed using essentially the same conditions and procedures reported previously [31]. Briefly, HCT116 cells expressing Raichu-Rac1 (FRET Rac1 biosensor) were generated by transposon-mediated gene transfer as described previously [32]. Stable transformants were selected using a FACSAria cell sorter (BD Biosciences). Forty-eight hours after HCT116 cells expressing Raichu-Rac1 were transfected with Halo-control or Halo-ARHGAP11A vector, transfected cells were labeled with Halo-tag TMR ligand (Promega) according to the manufacturer's instructions. Cells were imaged with an inverted microscope (FLUOVIEW FV1000 system; Olympus, Tokyo, Japan) equipped with a 60× objective lens (Olympus). FRET and TMR ligand images were taken sequentially using two different setups. A 440-nm or 559-nm diode laser was used as the excitation laser for the FRET or TMR ligand, respectively. The following setups were used for imaging studies: FRET, DM405–440/515, SDM510; TMR ligand, DM405/488/559, BA575–675. After background subtraction, FRET ratio images were created using the MetaMorph software (Universal Imaging, West Chester, PA), and were visualized in the intensity-modulated display mode. In this display mode, eight colors from red to blue are used to represent the FRET ratio (YFP/CFP), with the intensity of each color indicating the mean intensity of YFP and CFP.

### 3D Matrigel culture

To analyze 3D epithelial morphogenesis in Matrigel (BD Biosciences), 40 μl of growth factor-reduced Matrigel was mounted on a round coverslip and incubated at 37°C for 30 min to solidify the gel. HCT116 cells (2×10^4^) suspended in 1 ml of growth medium containing 2% Matrigel (v/v) and 10 ng/ml EGF (R&D Systems, Minneapolis, MN, USA) were added to solid Matrigel and incubated for a further 48 h [33].

### Matrigel invasion assay

Equal numbers of Matrigel-coated and control (uncoated) inserts were prepared for each experiment. HCT116 cells (1.0×10^5^ cells/ml) were suspended in DMED medium containing EGF (10 ng/ml). Y27632 was added in some experiments. An aliquot of cell suspension (5.0×10^4^ cells in 0.5 ml) was added to each 24-well chamber. DMED containing 10% FBS and EGF was added to each well of the 24-well plate as a chemoattractant. After incubation of invasion plates at 37°C in a 5% CO_2_ atmosphere for 36 h, non-migrating cells were removed with a cotton swab. The migrated cells on the lower surface of the membrane were stained with Diff-Quik. Stained cells were counted in three fields in triplicate. Invasion ratio was calculated as follows: Invasion index  =  mean number of cells migrating through the Matrigel matrix insert membrane/mean number of cells migrating through the uncoated insert membrane.

### Intravital multiphoton imaging of cancer cells

Intravital imaging was performed using protocols described previously with modifications [7]. To create subcutaneous or colon tumor models, HCT116 cells were injected into the subcutaneous or cecum tissue of immunocompromised NOD/SCID mice 5 weeks before observation [12]. Mice were anesthetized with isoflurane (2.0%, vaporized in air). Tumor masses were observed with an inverted multiphoton microscope (A1R-MP, Nikon), driven by a Chameleon Vision II Ti:Sapphire laser (Coherent) tuned to 940 nm and inverted microscopes equipped with multi-immersion objectives (CFI-Plan-Fluor, 20X/N.A. 0.75, Nikon). To detect mAG (Fucci-green), mKO2 (Fucci-red), and second harmonic generation (SHG) emission signals, 500/50-nm, 561/50-nm, and 440/50-nm band-pass filters were used, respectively. Raw imaging data were processed with Imaris (Bitplane) with a Gaussian filter for noise reduction. Automatic 3D object tracking with Imaris Spots was aided by manual correction to retrieve spatial cell co-ordinates over time.

### Extravasation model of cancer cells

Under anesthesia, an arc-shaped incision was made in the abdominal skin of nude mice according to previous report [56]. The connective tissue between skin and abdominal wall was separated to free the skin flap without injuring the epigastrica cranialis artery and vein with 29G needle and 1 ml syringe. The skin flap was spread and fixed on the flat stand. A total of 1×10^5^ cancer cells embedded with solution containing matrigel (25 μl) and complete medium (25 μl) were injected into the epigastrica cranialis vein. The skin flap was sewed and closed until imaging. When the imaging was performed, the skin flap was re-opened, spread and fixed at the imaging stage for intravital imaging. The inside surface of the skin flap was imaged.

### *In vivo* siRNA treatment

HCT116 cells (5.0×10^6^) were injected into subcutaneous tissues, and the resulting tumors were treated with siRNAs targeted to ARHGAP11A (Table S6) or a scrambled control siRNA, together with atelocollagen (AteloGene, Koken), 1 week after implantation. A 0.2-ml volume of siRNA solution (30 μmol/L in 0.5% (v/v) atelocollagen) was injected directly into tumors. Injected siRNAs were shown to remain stable *in vivo* for at least 1 week when supported by atelocollagen [36], [37].

### Fucci-signal-based microarray analysis

Two implanted tumors of HCT116 cells expressing Fucci were excised from two NOD/SCID mice. Then, each tumor was minced with a razor blade and treated with dispase and collagenase. Each sample was processed by a pipetting procedure and DNase treatment. Each sample was sorted into Fucci-green and -red cells. 1.0–2.0×10^5^ cells were collected from each sample. mRNA (600–1500 ng in 30 μl of distilled water) was extracted from each sample using an RNeasy mRNA purification kit (Qiagen). Microarray analysis was performed as described previously [57]. The fold-change values shown in the figures and tables are ratios of normalized values.

### Clinical sample microarrays

Tissues from 74 colorectal cancers and five normal colorectal tissue samples were obtained during surgery at Kyushu University Hospital, Beppu and its affiliated hospitals. Written informed consent was obtained from all patients and the study protocol was approved by the local ethics committee. Resected cancer tissues were sectioned and sampled by laser capture microdissection (LMD6000, Leica) [38], and microarray analyses were performed as described previously [35]. Primers used for qPCR analyses to confirm the expression of key molecules are listed in Table S7).

### Statistical analysis

Two-tailed Mann–Whitney U-tests, Kruskal–Wallis tests, and Dunnett's test were used to calculate *p*-values for skewed distributions. For Gaussian-like distributions, two-tailed Student's *t*-tests, one-way ANOVA, and Bonferroni's multiple comparison tests were used. Two-way ANOVA was used for implanted tumor size analysis.

### Accession numbers

Microarray data from Fucci-green/red HCT116 and from human colorectal cancer specimens are available through the NCBI GEO database [accession numbers: GSE34940 (for Fucci) and GSE35279 (for human sample)]. Datasets used for reanalysis were listed in Table S8.

## Supporting Information

Figure S1 Spatial distribution of G1 and S/G2/M cells in inoculated tumors. A human cancer cell line, HT1080, expressing Fucci was inoculated into the mesentery (A) and colon wall (B) of NOD/SCID mice. Four weeks after inoculation, Frozen sections of these tumors (n = 3) were observed under a confocal microscope (Nikon A1R). Green and red cells were enumerated using NIS-Elements (Nikon) with the manual assist function. Green to red cell ratios were calculated in both marginal and central regions of tumors. The ratio in the marginal area was significantly higher than that in the central area. Scale bars represent 100 μm. Data represent the means ± s.e.m.
(TIF)

Figure S2 Immunohistological analyses of a human colon cancer specimen stained with an anti-GMNN antibody. A representative entire image (A) and magnified views of non-tumor mucosa (B), tumor tissue around invasion areas (arrowheads) (C), and tumor tissue around the center of a tumor (asterisk) (D). Scale bars represent 1 cm (A) and 50 μm (B–D).
(TIF)

Figure S3 Time-courses of tracking velocities of cells during an extended period of intravital imaging. Velocities of Fucci-green and -red HCT116 cells were tracked with the Imaris software (Bitplane). Cell tracking velocities of Fucci-green and -red HCT116 cells were plotted. Over an extended period of time (∼150 min), mean tracking velocities were essentially unchanged.
(TIF)

Figure S4 Dynamic visualization of cell cycle progression. G1 (Fucci-red) cells were sorted from Fucci-bearing HCT116 cells using a FACSAria cell sorter (BD Biosciences). Time-lapse images of sorted G1 cells cultured in vitro taken using a confocal microscope (Nikon A1R). Fucci-green (mAG2) and red (mKO2) were excited by 488-nm and 561-nm laser lines, respectively. Band path filters (550/50 nm and 590/50 nm) were used for detection of mAG and mKO2. Fucci-red cells changed to Fucci-green cells in a time-dependent manner (A). Numbers of cells in the S/G2/M (green) and G1 (red) phases were counted using Imaris (Bitplane) (n = 8). There was significant interaction between cell numbers and time (two-way ANOVA, p<0.0001)
(TIF)

Figure S5 Cell cycle-dependent expression of ARHGAP11A in HeLa cells. Fucci-expressing HeLa cells were sorted into green and red cells (see the method for analysis of Fucci-expressing HCT116). mRNA and protein expression of ARHGAP11A were evaluated by qPCR (left) and Western blotting (right), respectively, and showed the cell cycle-dependent expression of this molecule in HeLa cells.
(TIF)

Figure S6 ARHGAP11A expression in a non-cancer cell line and normal tissues. (A) Western blotting analysis of ARHGAP11A expression in non-cancerous Fucci-expressing HEK293 cells. Cell cycle-dependent expression of ARHGAP11A was detected in HEK293 cells, and was synchronized with the expression of cyclin A and cyclin B1. (B) A representative image of normal colon mucosa stained with anti-ARHGAP11A antibody. Normal epithelial cells in the crypts, which are considered to be relatively proliferative (arrowheads), were stained modestly. The scale bar represents 100 μm.
(TIF)

Figure S7 ARHGAP11A suppressed the phosphorylation of MLC2. Immunocytochemical analysis of HCT116 (*left*) and HeLa (*right*) cells transfected with Halo-ARHGAP11A. Expression levels of phosphorylated myosin light chain 2 (pMLC2) (*green*) and F-actin (*red*) were reduced (*arrow*) in cells overexpressing Halo-ARHGAP11A (*blue*). The scale bars represent 20 μm.
(TIF)

Figure S8 Counter-activation of Rac1 in ARHGAP11A-expressing HeLa cells. (A) Rac1 activity in HeLa cells overexpressing Halo-ARHGAP11A. Representative images of HeLa cells overexpressing Halo-control (left) and Halo-ARHGAP11A (right). Scale bars represent 10 μm. (B) Quantification of FRET ratios of Halo-control (n = 51) and Halo-ARHGAP11A (n = 63). Error bars represent the s.e.m.
(TIF)

Figure S9 Integration of fluorescently labeled siRNAs against ARHGAP11A into cancer cells by *in vivo* siRNA treatment. One week after HCT116 cells expressing DsRed were inoculated into subcutaneous tissues, a FAM-labeled siRNA specific for ARHGAP11A (upper) and a non-labeled siRNA for ARHGAP11A (lower) were injected into the tissues surrounding tumors with atelocollagen. Three days later, the tumors were excised. Frozen tumor sections were visualized using a confocal microscope (Nikon A1). DAPI (blue), FAM (green) and DsRed (red).
(TIF)

Figure S10 Immunohistochemical detection of ARHGAP11A in human colon cancer samples. Paraffin sections were stained with anti-ARHGAP11A antibody. The upper and lower parts represent the luminal and serosal sides, respectively. Marginal ‘invading’ areas (*arrowheads*) in the tumor were preferentially stained compared to the central region. The scale bar represents 1 cm.
(TIF)

Figure S11 Correlations of microarray and qPCR data for human clinical samples. Microarray data were verified by quantitative real-time RT-PCR, performed using a LightCycler 480 System (Roche Applied Science) with a LightCycler 480 Probes Master kit (Roche Applied Science), according to a previous report. The mRNA expressions of five sample genes (*Arhgap11a* (a), *Geminin* (b), *Hmgb2* (c), *Opi5* (d), and *Top2a*(e)) were quantified by qPCR in 24 randomly selected clinical samples (the primers used are listed in Table S7). Correlations between relative levels in microarray and qPCR analyses were determined. The graphs show regression lines (solid line) and 95% confidence intervals (break line). The correlation coefficients for *Arhgap11a, Geminin, Hmgb2, Opi5*, and *Top2*a were 0.5154, 0.5930, 0.4559, 0.4258, and 0.3920, respectively.
(TIF)

Figure S12 Analyses of ARHGAP family protein expression in primary human colon cancer datasets. The correlation between the ARHGAP family and human colon cancer was determined using the NextBio data mining framework (http://www.nextbio.com). Nine previous microarray studies and our own findings, in terms of the comparison of human primary colon cancers and normal tissues, were included. Fold changes compared to normal tissues are shown in each graph. ARHGAP11A fold changes were elevated in 8 of 10 studies. Gene Expression Omnibus (GEO) accession numbers (NextBio ID) for these studies were: 1, our data; 2, GSE10972_1; 3, GSE20916_3; 4, GSE28000_GPL4133_2; 5, GSE21815_1; 6, GSE23878_1; 7, GSE18105_1; 8, GSE22598_1; 9, GSE25070_1; and 10, GSE31279_1 (Table S8).
(TIF)

Figure S13 Analyses of ARHGAP11A in primary human cancer datasets. The correlations between ARHGAP11A and various human cancers were determined using the NextBio data mining framework (http://www.nextbio.com). Studies comparing human cancers and normal tissues were selected. Fold changes compared to normal tissues are shown in the graphs. GEO accession numbers (NextBio ID) for these studies are shown in Table S8.
(TIF)

Figure S14 A computational method for measurement of F-actin intensity and focal adhesions. (A) Transfected cells (*arrowheads*) were labeled with Halo-Tag Oregon Green ligand (*green*). F-actin was labeled with Alexa 568-phaloidin (*red*). The anti-paxillin antibody (mouse monoclonal) was labeled with an Alexa 633-conjugated anti-mouse IgG antibody (*blue*). Regions containing transfected cells (*green*) were selected automatically and their areas (*upper right*) were measured using NIS-Elements. Then, the mean intensity of F-actin fluorescence in the whole cell area was measured (*lower left*) using the “Automatic Measurement” function in NIS-Elements. The focal adhesion area was defined as 2 μm^2^< area <50 μm^2^ and 400< fluorescent intensity of paxillin <2000. Focal adhesions were enumerated (lower left) using the “Object Counter” function in NIS-Elements (NIKON). Scale bars represent 20 μm.
(TIF)

Movie S1 Intravital multiphoton imaging of Fucci-expressing HCT116 inoculated subcutaneously in NOD/SCID mice. Sequential images in the same visual field were acquired. Some Fucci-green cells were seen to actively invading into interstitium. Fucci-green (mAG^+^), red (mKO2^+^) HCT116 cancer cells and collagen fibers in interstitium (visualized using second harmonic imaging) are shown as green, red, and blue, respectively. Scale bar, 100 μm. Playback speed is 300x.
(MP4)

Movie S2 Intravital multiphoton imaging of extravasating Fucci-expressing HCT116 cells. Sequential images in the same visual field were acquired. Fucci-green (mAG^+^), red (mKO2^+^) HCT116 cancer cells and perivascular collagen fibers (visualized using second harmonic imaging) are shown as green, red, and blue, respectively. Scale bar, 100 μm. Playback speed is 900x.
(MP4)

Movie S3 Intravital multiphoton imaging of Fucci-expressing HCT116 and tracking of cellular velocity in Fucci-green and red cells. Sequential images in the same visual field were acquired. Fucci-green and red positive cells were respectively tracked automatically by Imaris Spots. Fucci-green (mAG^+^), red (mKO2^+^) HCT116 cancer cells and collagen fibers in interstitium (visualized using second harmonic imaging) are shown as green, red, and blue, respectively. Scale bar, 100 μm. Playback speed is 1500x.
(MP4)

Movie S4 Intravital multiphoton imaging of control and ARHGAP11A-knockdown HCT116 cells. Sequential images in the same visual field were acquired. Control-EGFP^+^ and ARHGAP11A-knockdown-DsRed2^+^ HCT116 cells are shown as green and red, respectively. Scale bar, 50 μm. Playback speed is 900x.
(MP4)

Table S1 Top 50 genes up-regulated in S/G2/M phase in HCT116 expressing Fucci.
(DOCX)

Table S2 Primer pairs used for real-time qPCR for verifying Fucci microarray data. The expected molecular weight in base pairs (b.p.) is indicated.
(DOCX)

Table S3 The list of antibodies and their applications.
(DOCX)

Table S4 The list of sequences of shRNAs obtained from Sigma-Aldrich.
(DOCX)

Table S5 Primer pairs used to amplify a region of the Arhgap11a promoter. The expected molecular weight in base pairs (b.p.) is indicated.
(DOCX)

Table S6 The list of sequences of siRNAs duplex.
(DOCX)

Table S7 Primer pairs used for real-time qPCR for verifying microarray data with human colon cancer specimens. Primer sequences corresponding to universal probe libraries (UPL) and the expected molecular weight in base pairs (b.p.) are indicated.
(DOCX)

Table S8 The list of internal ID in Nextbio for re-analyses of RhoGAP expression.
(DOCX)
