# Supplementary figures and images for: Cell Cycle-Dependent Rho GTPase Activity Dynamically Regulates Cancer Cell Motility and Invasion In Vivo
Source: PLoS One. 2013 Dec 30;8(12):e83629. doi: 10.1371/journal.pone.0083629 (PMC3875446; doi:10.1371/journal.pone.0083629)

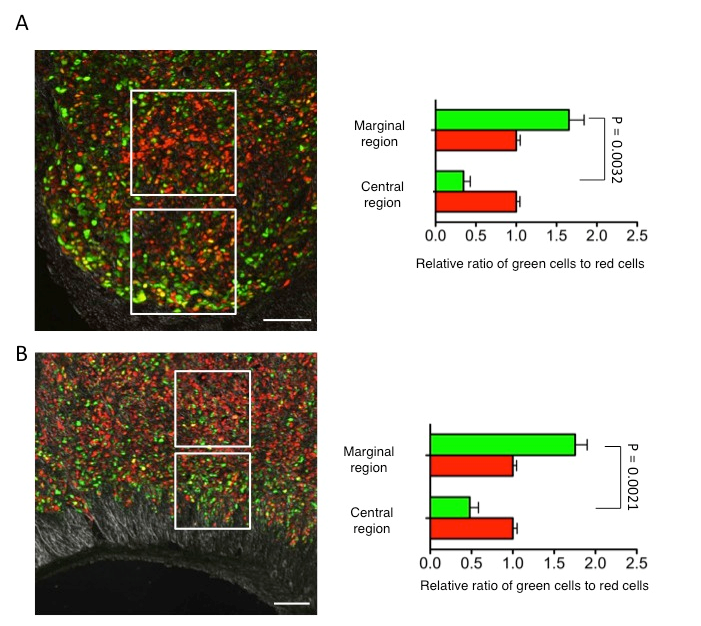

Supplement: Figure S1 — Spatial distribution of G1 and S/G2/M cells in inoculated tumors. A human cancer cell line, HT1080, expressing Fucci was inoculated into the mesentery (A) and colon wall (B) of NOD/SCID mice. Four weeks after inoculation, Frozen sections of these tumors (n = 3) were observed under a confocal microscope (Nikon A1R). Green and red cells were enumerated using NIS-Elements (Nikon) with the manual assist function. Green to red cell ratios were calculated in both marginal and central regions of tumors. The ratio in the marginal area was significantly higher than that in the central area. Scale bars represent 100 μm. Data represent the means ± s.e.m. (TIF) [file pone.0083629.s001.tif]

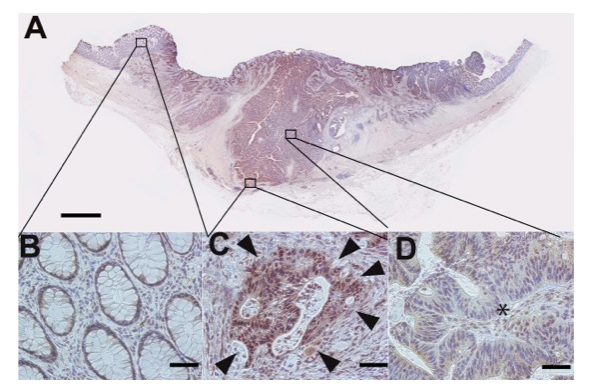

Supplement: Figure S2 — Immunohistological analyses of a human colon cancer specimen stained with an anti-GMNN antibody. A representative entire image (A) and magnified views of non-tumor mucosa (B), tumor tissue around invasion areas (arrowheads) (C), and tumor tissue around the center of a tumor (asterisk) (D). Scale bars represent 1 cm (A) and 50 μm (B–D). (TIF) [file pone.0083629.s002.tif]

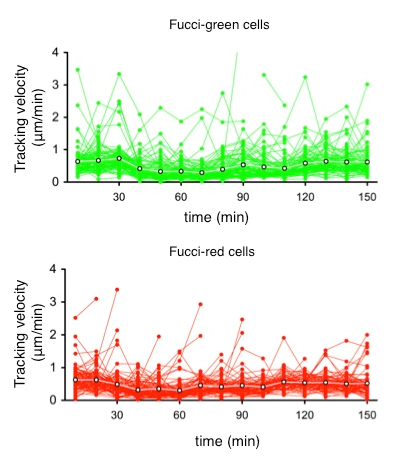

Supplement: Figure S3 — Time-courses of tracking velocities of cells during an extended period of intravital imaging. Velocities of Fucci-green and -red HCT116 cells were tracked with the Imaris software (Bitplane). Cell tracking velocities of Fucci-green and -red HCT116 cells were plotted. Over an extended period of time (∼150 min), mean tracking velocities were essentially unchanged. (TIF) [file pone.0083629.s003.tif]

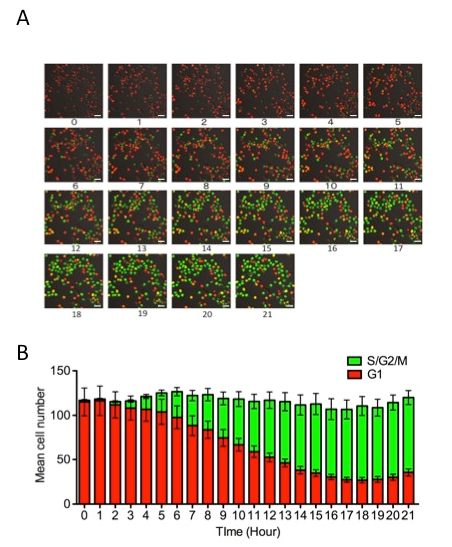

Supplement: Figure S4 — Dynamic visualization of cell cycle progression. G1 (Fucci-red) cells were sorted from Fucci-bearing HCT116 cells using a FACSAria cell sorter (BD Biosciences). Time-lapse images of sorted G1 cells cultured in vitro taken using a confocal microscope (Nikon A1R). Fucci-green (mAG2) and red (mKO2) were excited by 488-nm and 561-nm laser lines, respectively. Band path filters (550/50 nm and 590/50 nm) were used for detection of mAG and mKO2. Fucci-red cells changed to Fucci-green cells in a time-dependent manner (A). Numbers of cells in the S/G2/M (green) and G1 (red) phases were counted using Imaris (Bitplane) (n = 8). There was significant interaction between cell numbers and time (two-way ANOVA, p<0.0001) (TIF) [file pone.0083629.s004.tif]

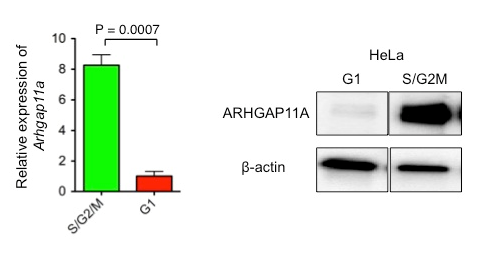

Supplement: Figure S5 — Cell cycle-dependent expression of ARHGAP11A in HeLa cells. Fucci-expressing HeLa cells were sorted into green and red cells (see the method for analysis of Fucci-expressing HCT116). mRNA and protein expression of ARHGAP11A were evaluated by qPCR (left) and Western blotting (right), respectively, and showed the cell cycle-dependent expression of this molecule in HeLa cells. (TIF) [file pone.0083629.s005.tif]

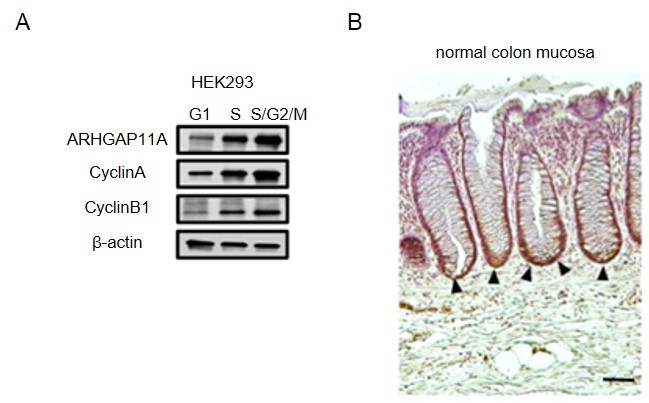

Supplement: Figure S6 — ARHGAP11A expression in a non-cancer cell line and normal tissues. (A) Western blotting analysis of ARHGAP11A expression in non-cancerous Fucci-expressing HEK293 cells. Cell cycle-dependent expression of ARHGAP11A was detected in HEK293 cells, and was synchronized with the expression of cyclin A and cyclin B1. (B) A representative image of normal colon mucosa stained with anti-ARHGAP11A antibody. Normal epithelial cells in the crypts, which are considered to be relatively proliferative (arrowheads), were stained modestly. The scale bar represents 100 μm. (TIF) [file pone.0083629.s006.tif]

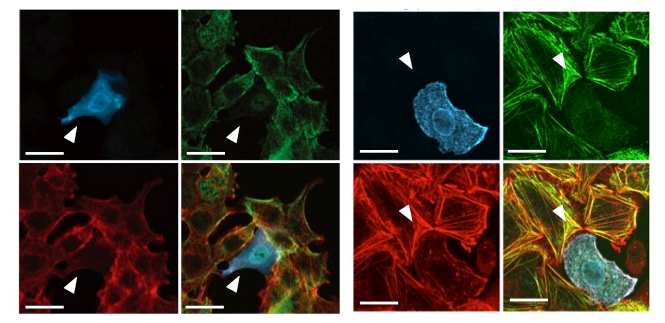

Supplement: Figure S7 — ARHGAP11A suppressed the phosphorylation of MLC2. Immunocytochemical analysis of HCT116 (left) and HeLa (right) cells transfected with Halo-ARHGAP11A. Expression levels of phosphorylated myosin light chain 2 (pMLC2) (green) and F-actin (red) were reduced (arrow) in cells overexpressing Halo-ARHGAP11A (blue). The scale bars represent 20 μm. (TIF) [file pone.0083629.s007.tif]

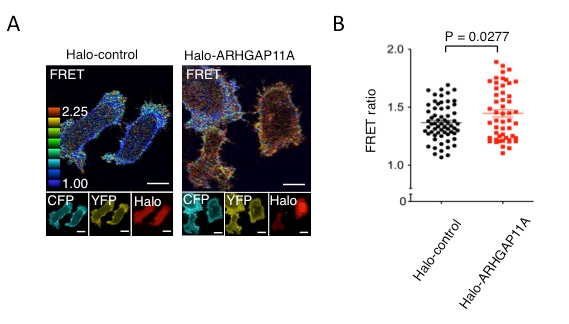

Supplement: Figure S8 — Counter-activation of Rac1 in ARHGAP11A-expressing HeLa cells. (A) Rac1 activity in HeLa cells overexpressing Halo-ARHGAP11A. Representative images of HeLa cells overexpressing Halo-control (left) and Halo-ARHGAP11A (right). Scale bars represent 10 μm. (B) Quantification of FRET ratios of Halo-control (n = 51) and Halo-ARHGAP11A (n = 63). Error bars represent the s.e.m. (TIF) [file pone.0083629.s008.tif]

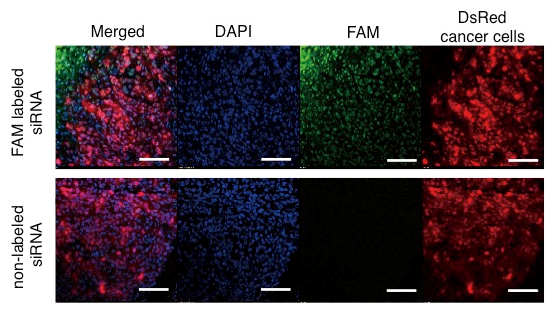

Supplement: Figure S9 — Integration of fluorescently labeled siRNAs against ARHGAP11A into cancer cells by in vivo siRNA treatment. One week after HCT116 cells expressing DsRed were inoculated into subcutaneous tissues, a FAM-labeled siRNA specific for ARHGAP11A (upper) and a non-labeled siRNA for ARHGAP11A (lower) were injected into the tissues surrounding tumors with atelocollagen. Three days later, the tumors were excised. Frozen tumor sections were visualized using a confocal microscope (Nikon A1). DAPI (blue), FAM (green) and DsRed (red). (TIF) [file pone.0083629.s009.tif]

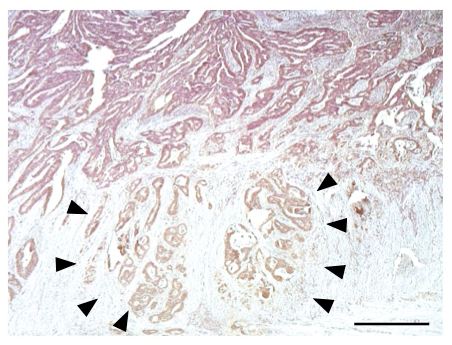

Supplement: Figure S10 — Immunohistochemical detection of ARHGAP11A in human colon cancer samples. Paraffin sections were stained with anti-ARHGAP11A antibody. The upper and lower parts represent the luminal and serosal sides, respectively. Marginal ‘invading’ areas (arrowheads) in the tumor were preferentially stained compared to the central region. The scale bar represents 1 cm. (TIF) [file pone.0083629.s010.tif]

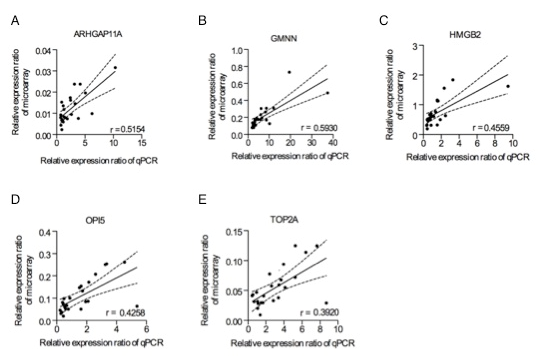

Supplement: Figure S11 — Correlations of microarray and qPCR data for human clinical samples. Microarray data were verified by quantitative real-time RT-PCR, performed using a LightCycler 480 System (Roche Applied Science) with a LightCycler 480 Probes Master kit (Roche Applied Science), according to a previous report. The mRNA expressions of five sample genes (Arhgap11a (a), Geminin (b), Hmgb2 (c), Opi5 (d), and Top2a(e)) were quantified by qPCR in 24 randomly selected clinical samples (the primers used are listed in Table S7). Correlations between relative levels in microarray and qPCR analyses were determined. The graphs show regression lines (solid line) and 95% confidence intervals (break line). The correlation coefficients for Arhgap11a, Geminin, Hmgb2, Opi5, and Top2a were 0.5154, 0.5930, 0.4559, 0.4258, and 0.3920, respectively. (TIF) [file pone.0083629.s011.tif]

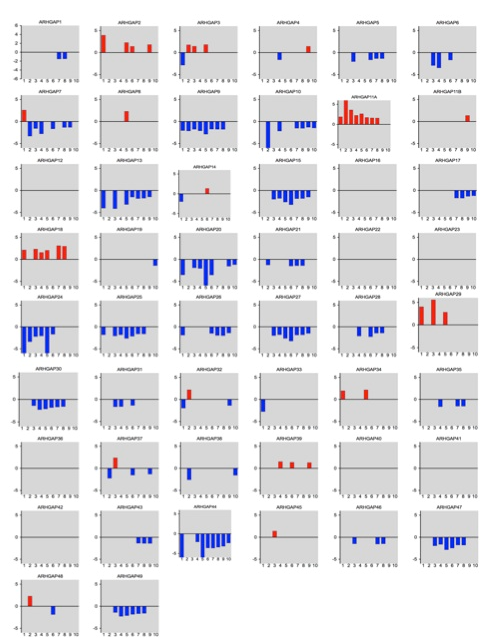

Supplement: Figure S12 — Analyses of ARHGAP family protein expression in primary human colon cancer datasets. The correlation between the ARHGAP family and human colon cancer was determined using the NextBio data mining framework (http://www.nextbio.com). Nine previous microarray studies and our own findings, in terms of the comparison of human primary colon cancers and normal tissues, were included. Fold changes compared to normal tissues are shown in each graph. ARHGAP11A fold changes were elevated in 8 of 10 studies. Gene Expression Omnibus (GEO) accession numbers (NextBio ID) for these studies were: 1, our data; 2, GSE10972_1; 3, GSE20916_3; 4, GSE28000_GPL4133_2; 5, GSE21815_1; 6, GSE23878_1; 7, GSE18105_1; 8, GSE22598_1; 9, GSE25070_1; and 10, GSE31279_1 (Table S8). (TIF) [file pone.0083629.s012.tif]

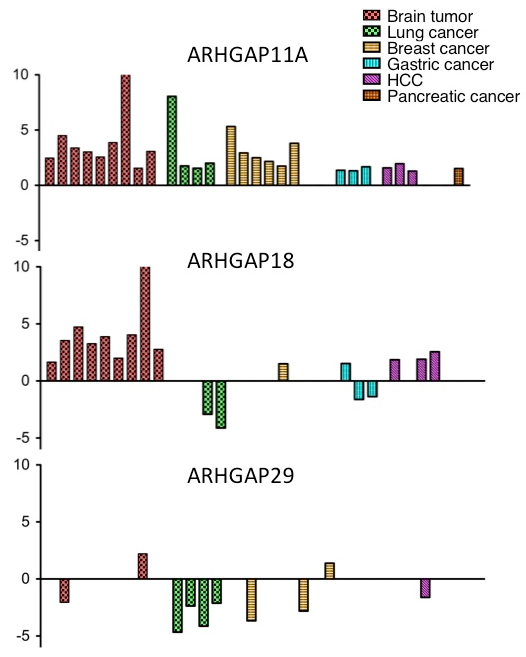

Supplement: Figure S13 — Analyses of ARHGAP11A in primary human cancer datasets. The correlations between ARHGAP11A and various human cancers were determined using the NextBio data mining framework (http://www.nextbio.com). Studies comparing human cancers and normal tissues were selected. Fold changes compared to normal tissues are shown in the graphs. GEO accession numbers (NextBio ID) for these studies are shown in Table S8. (TIF) [file pone.0083629.s013.tif]

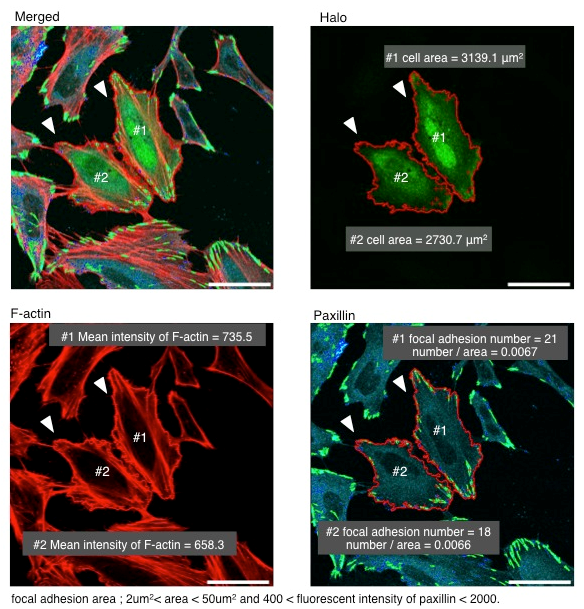

Supplement: Figure S14 — A computational method for measurement of F-actin intensity and focal adhesions. (A) Transfected cells (arrowheads) were labeled with Halo-Tag Oregon Green ligand (green). F-actin was labeled with Alexa 568-phaloidin (red). The anti-paxillin antibody (mouse monoclonal) was labeled with an Alexa 633-conjugated anti-mouse IgG antibody (blue). Regions containing transfected cells (green) were selected automatically and their areas (upper right) were measured using NIS-Elements. Then, the mean intensity of F-actin fluorescence in the whole cell area was measured (lower left) using the “Automatic Measurement” function in NIS-Elements. The focal adhesion area was defined as 2 μm2< area <50 μm2 and 400< fluorescent intensity of paxillin <2000. Focal adhesions were enumerated (lower left) using the “Object Counter” function in NIS-Elements (NIKON). Scale bars represent 20 μm. (TIF) [file pone.0083629.s014.tif]
