# Supplementary material for: Cell Cycle-Dependent Rho GTPase Activity Dynamically Regulates Cancer Cell Motility and Invasion In Vivo
Source: PLoS One. 2013 Dec 30;8(12):e83629. doi: 10.1371/journal.pone.0083629 (PMC3875446; doi:10.1371/journal.pone.0083629)
Supplement: Table S1 — Top 50 genes up-regulated in S/G2/M phase in HCT116 expressing Fucci. (DOCX) [file pone.0083629.s019.docx]

| Rank | FC | Symbol | Description |
| --- | --- | --- | --- |
| 1 | 30.1 | CENPA | centromere protein A |
| 2 | 26.4 | A_24_P221285 |  |
| 3 | 26 | PRR11 | proline rich 11 |
| 4 | 19.9 | CKAP2L | cytoskeleton associated protein 2-like |
| 5 | 18.9 | ARHGAP11A | Rho GTPase activating protein 11A |
| 6 | 17.2 | SPC25 | SPC25, NDC80 kinetochore complex component, homolog (S. cerevisiae) |
| 7 | 17 | MKI67 | antigen identified by monoclonal antibody Ki-67 |
| 8 | 16 | NEIL3 | nei endonuclease VIII-like 3 (E. coli) |
| 9 | 16 | CENPI | centromere protein I |
| 10 | 15.9 | CDCA2 | cell division cycle associated 2 |
| 11 | 15 | SKA1 | spindle and kinetochore associated complex subunit 1 |
| 12 | 14.9 | ARHGAP11A | Rho GTPase activating protein 11A |
| 13 | 14.9 | PIF1 | PIF1 5'-to-3' DNA helicase homolog (S. cerevisiae) |
| 14 | 14.8 | HMMR | hyaluronan-mediated motility receptor (RHAMM) |
| 15 | 14.6 | ASPM | asp (abnormal spindle) homolog, microcephaly associated (Drosophila) |
| 16 | 14.4 | TOP2A | topoisomerase (DNA) II alpha 170kDa |
| 17 | 14.3 | NDC80 | NDC80 homolog, kinetochore complex component (S. cerevisiae) |
| 18 | 14.3 | CDC2 | cell division cycle 2, G1 to S and G2 to M |
| 19 | 14.3 | KIF14 | kinesin family member 14 |
| 20 | 13.9 | KIF23 | kinesin family member 23 |
| 21 | 13.8 | CDCA8 | cell division cycle associated 8 |
| 22 | 13.8 | DLGAP5 | discs, large (Drosophila) homolog-associated protein 5 |
| 23 | 12.4 | CDC25C | cell division cycle 25 homolog C (S. pombe) |
| 24 | 12.2 | NEK2 | NIMA (never in mitosis gene a)-related kinase 2 |
| 25 | 12.2 | BUB1 | budding uninhibited by benzimidazoles 1 homolog (yeast) |
| 26 | 12.1 | CASC5 | cancer susceptibility candidate 5 |
| 27 | 12.1 | A_24_P50328 |  |
| 28 | 12 | ASPM | asp (abnormal spindle) homolog, microcephaly associated (Drosophila) |
| 29 | 12 | CENPI | centromere protein I |
| 30 | 12 | CDC2 | cell division cycle 2, G1 to S and G2 to M |
| 31 | 11.9 | KIF15 | kinesin family member 15 |
| 32 | 11.9 | SHCBP1 | SHC SH2-domain binding protein 1 |
| 33 | 11.6 | FAM72D | family with sequence similarity 72, member D |
| 34 | 11.5 | A_24_P306896 |  |
| 35 | 11.4 | TTK | TTK protein kinase |
| 36 | 11.4 | FAM64A | family with sequence similarity 64, member A |
| 37 | 11.3 | NEK2 | NIMA (never in mitosis gene a)-related kinase 2 |
| 38 | 11.3 | CCNA2 | cyclin A2 |
| 39 | 11.2 | FAM83D | family with sequence similarity 83, member D |
| 40 | 11.2 | CEP55 | centrosomal protein 55kDa |
| 41 | 11 | ARHGAP11A | Rho GTPase activating protein 11A |
| 42 | 11 | CENPF | centromere protein F, 350/400ka (mitosin) |
| 43 | 11 | NUF2 | NUF2, NDC80 kinetochore complex component, homolog (S. cerevisiae) |
| 44 | 10.9 | NDC80 | NDC80 homolog, kinetochore complex component (S. cerevisiae) |
| 45 | 10.7 | CASC5 | cancer susceptibility candidate 5 |
| 46 | 10.7 | SKA3 | spindle and kinetochore associated complex subunit 3 |
| 47 | 10.7 | ESCO2 | establishment of cohesion 1 homolog 2 (S. cerevisiae) |
| 48 | 10.6 | RRM2 | ribonucleotide reductase M2 |
| 49 | 10.6 | CENPF | centromere protein F, 350/400ka (mitosin) |
| 50 | 10.5 | KIF11 | kinesin family member 11 |

FC: fold change
