# Supplementary material for: Cell Cycle-Dependent Rho GTPase Activity Dynamically Regulates Cancer Cell Motility and Invasion In Vivo
Source: PLoS One. 2013 Dec 30;8(12):e83629. doi: 10.1371/journal.pone.0083629 (PMC3875446; doi:10.1371/journal.pone.0083629)
Supplement: Table S2 — Primer pairs used for real-time qPCR for verifying Fucci microarray data. The expected molecular weight in base pairs (b.p.) is indicated. (DOCX) [file pone.0083629.s020.docx]

|  | Forward (5’-3’) | Reverse (5’-3’) | b.p. |
| --- | --- | --- | --- |
| *Arhgap11a* | GCAGGTGTGCCAAGGCGAAGT | TGCAAGTCGCCAACCAACACTTTCA | 210 |
| *Gapdh* | TGTTGCCATCAATGACCCCTT | CTCCACGACGTACTCAGCG | 202 |
