# Supplementary material for: Cell Cycle-Dependent Rho GTPase Activity Dynamically Regulates Cancer Cell Motility and Invasion In Vivo
Source: PLoS One. 2013 Dec 30;8(12):e83629. doi: 10.1371/journal.pone.0083629 (PMC3875446; doi:10.1371/journal.pone.0083629)
Supplement: Table S3 — The list of antibodies and their applications. (DOCX) [file pone.0083629.s021.docx]

| Primary antibody | Company | Catalog ID | Application(s) and working dilution(s) |
| --- | --- | --- | --- |
| Anti-phospho-myosin light chain 2 (Thr18/Ser19) | Cell Signaling | #3674 | IF 1:200 |
| Anti-RhoA | Cell Signaling | #2117 | IP 1:1000 |
| Anti-RhoB | Cell Signaling | #2098 | IP 1:1000 |
| Anti-RhoC | Cell Signaling | #3430 | IP 1:1000 |
| Anti-Rac1 | Cell Signaling | #2465 | IP 1:1000 |
| Anti-Cdc42 | Cell Signaling | #2462 | IP 1:1000 |
| Anti-paxillin | BD | #2542 | IF 1:500 |
| Anti-ARHGAP11A | Abcam | #ab113261 | WB 1:1000 |
| Anti-ARHGAP11A | Sigma-Aldrich | HPA040830 | IHC, IF 1:200 |
| Anti-CyclinA | Santa Cruz | sc-751 | WB 1:200 |
| Anti-CyclinB1 | Cell Signaling | #4138 | WB 1:1000 |
| Anti-cdc2 | Cell Signaling | #9112 | WB 1:1000 |
| Anti-phospho-cdc2 | Cell Signaling | #9111 | WB 1:1000 |
| Anti-Rb | Cell Signaling | #9309 | WB 1:1000 |
| Anti-phospho Rb | Cell Signaling | #9308 | WB 1:1000 |
| Anti-beta actin | Santa Cruz | sc-47778 | WB 1:1000 |
| Anti-Halo Tag | Promega | G928A | WB 1:200, IF: 1:50 |
| Anti-E2F1 | Santa Cruz | sc-193x | CHIP 1μg/200μg |
| Normal rabbit IgG | Santa Cruz | sc-2027 | CHIP 1μg/200μg |
