# Supplementary material for: Cell Cycle-Dependent Rho GTPase Activity Dynamically Regulates Cancer Cell Motility and Invasion In Vivo
Source: PLoS One. 2013 Dec 30;8(12):e83629. doi: 10.1371/journal.pone.0083629 (PMC3875446; doi:10.1371/journal.pone.0083629)
Supplement: Table S4 — The list of sequences of shRNAs obtained from Sigma-Aldrich. (DOCX) [file pone.0083629.s022.docx]

| shRNA target | Sequence strand (5’-3’) | Catalog ID |
| --- | --- | --- |
| ARHGAP11A #1 | CCGGCGGTATCAGTTCACATCGATACTCGAGTATCGATGTGAACTGATACCGTTTTTG | TRCN0000047281 |
| ARHGAP11A #2 | CCGGCCTTCTATTACACCTCAAGAACTCGAGTTCTTGAGGTGTAATAGAAGGTTTTTG | TRCN0000047282 |
| Control sh | CCGGCAACAAGATGAAGAGCACCAACTCGAGTTGGTGCTCTTCATCTTGTTGTTTTT | SHC002 |
