# Supplementary material for: Cell Cycle-Dependent Rho GTPase Activity Dynamically Regulates Cancer Cell Motility and Invasion In Vivo
Source: PLoS One. 2013 Dec 30;8(12):e83629. doi: 10.1371/journal.pone.0083629 (PMC3875446; doi:10.1371/journal.pone.0083629)
Supplement: Table S5 — Primer pairs used to amplify a region of the Arhgap11a promoter. The expected molecular weight in base pairs (b.p.) is indicated. (DOCX) [file pone.0083629.s023.docx]

|  | Forward (5’-3’) | Reverse (5’-3’) | b.p. |
| --- | --- | --- | --- |
| ARHGAP11A promoter | CAGATGTGGAGCGCTGTTTCGCG | GCGTTCACTGAAGCCAAGCCGT | 139 |
