# Supplementary material for: Cell Cycle-Dependent Rho GTPase Activity Dynamically Regulates Cancer Cell Motility and Invasion In Vivo
Source: PLoS One. 2013 Dec 30;8(12):e83629. doi: 10.1371/journal.pone.0083629 (PMC3875446; doi:10.1371/journal.pone.0083629)
Supplement: Table S6 — The list of sequences of siRNAs duplex. (DOCX) [file pone.0083629.s024.docx]

| siRNA target | Sense (5’-3’) | Anti-sense (5’-3’) |
| --- | --- | --- |
| ARHGAP11A #1 | UCUUUAUGGCUGAUUUUAGGATT | UCCUAAAAUCAGCCAUAAAGATT |
| ARHGAP11A #2 | UAUUGAAGAGAUUACUUGGCATT | UGCCAAGUAAUCUCUUCAAUATT |
| Scrambled | AUCCGCGCGAUAGUACGUATT | UACGUACUAUCGCGCGGAUTT |
