# Supplementary material for: Cell Cycle-Dependent Rho GTPase Activity Dynamically Regulates Cancer Cell Motility and Invasion In Vivo
Source: PLoS One. 2013 Dec 30;8(12):e83629. doi: 10.1371/journal.pone.0083629 (PMC3875446; doi:10.1371/journal.pone.0083629)
Supplement: Table S7 — Primer pairs used for real-time qPCR for verifying microarray data with human colon cancer specimens. Primer sequences corresponding to universal probe libraries (UPL) and the expected molecular weight in base pairs (b.p.) are indicated. (DOCX) [file pone.0083629.s025.docx]

|  | Forward (5’-3’) | Reverse (5’-3’) | UPL No. | b.p. |
| --- | --- | --- | --- | --- |
| *Arhgap11a* | TCGAAGATCTCTGCGTTTGA | ATTGACACCAGAACATCCATTTA | #53 | 73 |
| *Geminin* | AACTGGCAGAAGTAGCAGAACA | CCAGAGGTTCACCATTCAGTC | #53 | 72 |
| *Hmgb2* | TGAACAGAAAGCAGCTAAGCTAAA | CTTCTTCTTTGAGCCTGTTGG | #81 | 121 |
| *Opi5* | CTGGCTGCCTTGAGAGGT | TGATGCATTTACTATGGCTTTTG | #29 | 87 |
| *Top2a* | CAACATGCCAATTGAGTGAAA | GGACTTGGGCCTTAAACTTCA | #76 | 96 |
| *Gapdh* | GCCCAATACGACCAAATCC | AGCCACATCGCTCAGACAC | #60 | 115 |
