# Supplementary material for: Cell Cycle-Dependent Rho GTPase Activity Dynamically Regulates Cancer Cell Motility and Invasion In Vivo
Source: PLoS One. 2013 Dec 30;8(12):e83629. doi: 10.1371/journal.pone.0083629 (PMC3875446; doi:10.1371/journal.pone.0083629)
Supplement: Table S8 — The list of internal ID in Nextbio for re-analyses of RhoGAP expression. (DOCX) [file pone.0083629.s026.docx]

| Colon | Brain tumor | Lung tumor | Breast cancer |
| --- | --- | --- | --- |
| our data | GSE10878_1 | GSE17599_4 | GSE22820_1 |
| GSE10972_1 | GSE15209_2 | GSE5364_7 | GSE21997_GPL5325_12 |
| GSE20916_3 | TCGA-Agilent_4 | GSE18842_1 | GSE14999_1 |
| GSE28000_GPL4133_2 | TCGA-Agilent_1 | GSE19188_3 | GSE10886_1 |
| GSE21815_1 | TCGA-Agilent_5 |  | GSE20711_1 |
| GSE23878_1 | TCGA-Agilent_7 |  | GSE22358_1 |
| GSE18105_1 | GSE22866_1 |  | GSE31448_1 |
| GSE22598_1 | GSE15824_14 |  | GSE3165_GPL1390_1 |
| GSE25070_1 | GSE4290_3 |  |  |
| GSE31279_1 |  |  |  |

| Gastric cancer | Hepatocellular carcinoma | Pancreatic cancer |
| --- | --- | --- |
| GSE19826_2 | GSE6222_2 | GSE1542_11 |
| GSE13911_2 | GSE25097_2 | GSE19650_1 |
| GSE33651_1 | GSE6764 |  |
|  | GSE36411_3 |  |
